# Encoding of Sucrose's Palatability in the Nucleus Accumbens Shell and Its Modulation by Exteroceptive Auditory Cues

**DOI:** 10.3389/fnins.2018.00265

**Published:** 2018-05-04

**Authors:** Miguel Villavicencio, Mario G. Moreno, Sidney A. Simon, Ranier Gutierrez

**Affiliations:** ^1^Laboratory of Neurobiology of Appetite, Department of Pharmacology, Centro de Investigación y de Estudios Avanzados del Instituto Politécnico Nacional, Mexico City, Mexico; ^2^Department of Neurobiology, Duke University Medical Center, Durham, NC, United States

**Keywords:** sucrose palatability, auditory cues, taste, feeding, licking, obesity

## Abstract

Although the palatability of sucrose is the primary reason for why it is over consumed, it is not well understood how it is encoded in the nucleus accumbens shell (NAcSh), a brain region involved in reward, feeding, and sensory/motor transformations. Similarly, untouched are issues regarding how an external auditory stimulus affects sucrose palatability and, in the NAcSh, the neuronal correlates of this behavior. To address these questions in behaving rats, we investigated how food-related auditory cues modulate sucrose's palatability. The goals are to determine whether NAcSh neuronal responses would track sucrose's palatability (as measured by the increase in hedonically positive oromotor responses lick rate), sucrose concentration, and how it processes auditory information. Using brief-access tests, we found that sucrose's palatability was enhanced by exteroceptive auditory cues that signal the start and the end of a reward epoch. With only the start cue the rejection of water was accelerated, and the sucrose/water ratio was enhanced, indicating greater palatability. However, the start cue also fragmented licking patterns and decreased caloric intake. In the presence of both start and stop cues, the animals fed continuously and increased their caloric intake. Analysis of the licking microstructure confirmed that auditory cues (either signaling the start alone or start/stop) enhanced sucrose's oromotor-palatability responses. Recordings of extracellular single-unit activity identified several distinct populations of NAcSh responses that tracked either the sucrose palatability responses or the sucrose concentrations by increasing or decreasing their activity. Another neural population fired synchronously with licking and exhibited an enhancement in their coherence with increasing sucrose concentrations. The population of NAcSh's Palatability-related and Lick-Inactive neurons were the most important for decoding sucrose's palatability. Only the Lick-Inactive neurons were phasically activated by both auditory cues and may play a sentinel role monitoring relevant auditory cues to increase caloric intake and sucrose's palatability. In summary, we found that auditory cues that signal the availability of sucrose modulate its palatability and caloric intake in a task dependent-manner and had neural correlates in the NAcSh. These findings show that exteroceptive cues associated with feeding may enhance positive hedonic oromotor-responses elicited by sucrose's palatability.

## Introduction

In the last 30 years, the prevalence of worldwide obesity has nearly tripled (Bleich et al., 2008). One culprit of this epidemic is the overconsumption of sucrose (Popkin and Nielsen, 2003). Its highly attractive properties are mainly attributed to its hedonically positive sweet taste that elicits characteristic oro-motor responses such as facial expressions and appetitive ingestive responses (Davis, 1973; Pfaffmann and Norgren, 1977; Spector et al., 1998). Throughout the taste pathway sucrose also evokes concentration-dependent neural activity (Rolls, 1989; Stapleton et al., 2006; Chen et al., 2011; Roussin et al., 2012; Jezzini et al., 2013; Wu et al., 2015). Sucrose also alters the activity of the nucleus accumbens shell (NAcSh), a brain region involved in reward (Hajnal et al., 2004). That is, NAcSh neurons have been found to encode sucrose's palatability and its reward value (Taha and Fields, 2005).

Studies on the NAcSh have been performed relating its responses to the palatability of hedonically positive and negative tastants (Roitman et al., 2005; Krause et al., 2010). For example, Castro and Berridge (2014) found that opioids injected directly in the NAcSh enhance feeding and oromotor movements thereby reflecting increased palatability. Recordings from NAcSh have consistently revealed that the majority of neurons decreased their activity, whereas a smaller population were activated during consummatory licking (Roitman et al., 2005; Krause et al., 2010; Tellez et al., 2012). Nevertheless, there is still not a consensus regarding how NAcSh neurons track the palatability of sucrose (Roitman et al., 2005; Taha and Fields, 2005). In this regard, using a Pavlovian conditioning task, Roitman et al. (2005) found that NAcSh neurons were innately tuned for rewarding and aversive taste stimuli since they were inhibited by sucrose and activated by quinine, a bitter tastant. In contrast, Taha and Fields (2005) using a sucrose discrimination task have proposed just the opposite scenario; that is, the activated responses encode the palatability/reward aspects of sucrose, whereas the inactivated neurons initiate and sustain feeding. Thus, currently, there is little agreement regarding how NAcSh neurons track taste palatability. Importantly, the neuronal correlates of a behavioral task designed to measure taste palatability (Young and Trafton, 1964; Davis, 1973; Smith et al., 1992) in the NAcSh have not been characterized.

In a recent optogenetic study in the rat NAcSh, O'Connor et al. (2015) identified that the neurons that were inhibited by licking (Lick-Inactive neurons) to be medium spiny neurons expressing the D1 receptor (MSND1+). They found that optogenetic silencing prolonged sucrose feeding, even in the presence of a threatening auditory tone that could interrupt consumption. In contrast, their optogenetic stimulation stopped feeding. Their findings suggest that NAcSh neurons can play a “sentinel role” for feeding, by integrating auditory stimuli to authorize (or gate) consumption (Krause et al., 2010; O'Connor et al., 2015). However, the interaction between auditory cues, sucrose palatability, and NAcSh responses has not been fully explored. Although Taha and Fields (2005) used white noise to cue rats about when they could start licking for sucrose, they did not use a control task without auditory cues. Thus, it remains to be elucidated whether NAcSh neurons are responsive to both auditory and taste information in a manner that can modulate sucrose palatability.

To address these issues, we recorded neuronal activity from the NAcSh while rats performed one of three variants of a brief-access taste test used to measure palatability responses (Young and Trafton, 1964; Davis, 1973; Smith et al., 1992). One control test is where animals could only use taste/somatosensory information (Gustatory test) and two other variants that included either one or two auditory cues that signal the start and/or the end of a reward period (Start test and Start/Stop test, respectively). These behavioral studies revealed that the addition of taste-related auditory cues differentially enhanced, in a task-dependent manner, sucrose's palatability and caloric intake. Electrophysiological recordings from the NAcSh together with a novel “best-window” neuronal analysis uncovered a population of neurons with increasing or decreasing firing rates that correlated with the oromotor (lick rate) responses elicited by sucrose's palatability. These results revealed that NAcSh palatability-related neurons include a larger and more heterogeneous group than was previously assigned (Roitman et al., 2005; Taha and Fields, 2005). In addition, we found that the activity of the Palatability-related neurons could dynamically track the changes in oromotor responses elicited by sucrose over the course of the behavioral session. Another neuronal population tracked the sucrose concentration independent of the licking rate. Importantly, and in contrast to a previous study (Taha and Fields, 2005), we found for the first time that the firing rate of the Lick-Inactive neurons correlated with the sucrose's palatability. These neurons also were phasically activated when both the Start and Stop auditory cues signaled the start and end of the Reward epoch and thus they monitor the presence of oral chemical stimuli, auditory cues, and oromotor-palatability information to guide animals to initiate and terminate food intake. In summary, these data show that exteroceptive cues that signal tastant availability can enhance sucrose's caloric intake and palatability and that NAcSh neurons play an important role tracking sucrose's oromotor-palatability and its modulation by auditory cues.

## Materials and methods

### Subjects and surgery

Sprague-Dawley male rats (*n* = 30) weighing 300–350 g were individually housed and maintained at 21 ± 1°C and 12:12 h light/dark cycle (lights on 0700; and lights off 1900). Three groups of naïve subjects (each group *n* = 10) were implanted in the NAcSh with a homemade 16-channel electrode array using the following coordinates AP: 1.4 mm, ML: −1 mm, DV: −7.5 mm, from bregma (Tellez et al., 2012). For surgery, animals were anesthetized using an intraperitoneal injection of ketamine (90 mg/Kg)/xylazine (8 mg/Kg). After surgery, they received enrofloxacin (45 mg/Kg) for three additional days. After a 7-day recovery period, each group of rats were 22 h water-restricted (Gutierrez et al., 2010), and they were trained for 13 days in one of the three versions of a brief-access taste test (each session lasted 55 min), while extracellular single-unit recordings from the NAcSh were simultaneously performed. Each animal was always recorded at the same time of the day between 1700 to 1900 h (see below). Immediately, after every recording session, rats received 1 h and 5 min access to tap water at their home cages. This chronic water restriction protocol is a standard procedure that is necessary for rats to maintain high levels of motivation for operant-conditioning tasks (Hughes et al., 1994), without affecting their health (Rowland, 2007). Chow food diet (LabDiet 5008) was available *ad libitum* in their home cages.

To verify the location of electrodes, at the end of each experiment animals were sacrificed by an overdose of pentobarbital (150 mg/kg) and 50 μm brain slices were obtained and stained with cresyl-violet. The CINVESTAV Animal Care and Use Committee approved all animal procedures (see Methods and Supplementary Methods).

### Tastants

Taste stimuli consisted of deionized water and reagent-grade sucrose (Sigma-Aldrich, Mexico) dissolved in deionized water. We used four semi-logarithmically spaced sucrose concentrations: 3, 5.8, 10.7, and 20 wt. / vol.% (0.087, 0.168, 0.31, 0.58 M, respectively). All taste stimuli were prepared daily and used at room temperature.

### Behavior

**Figure 1:**
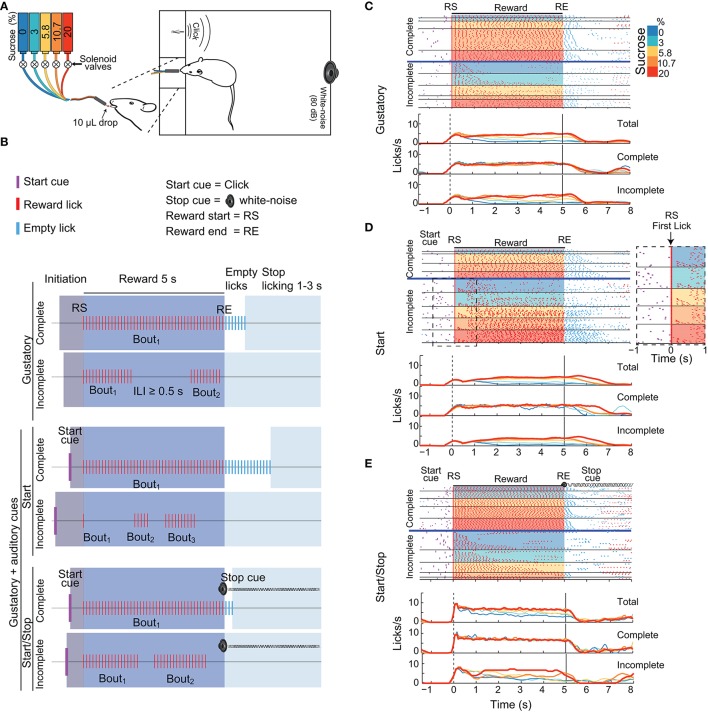
Three variants of the brief-access taste tests used in this study. **(A)** Schematic showing a rat in a behavioral box with a tastant delivery system. The box was equipped with a sipper that delivers, in each lick, a drop of either water or concentrations of sucrose (color-coded). Also seen are two instruments that produce auditory cues: a “click” and a white-noise cue. **(B)** Schematics of the structure of trials in the Gustatory (upper) and the two Gustatory + auditory (bottom) variants (Start and Start/Stop) of the brief-access tests. The depicted trials are colored as a function of four epochs: Initiation (gray), Reward (blue), Empty licks (white) and Stop licking (cyan). RS indicates the Reward Start, whereas RE shows the Reward End. All trials were classified as being either Complete (where animals lick continuously emitting one single bout) or Incomplete (where they pause licking at least once for an inter-lick interval (ILI) ≥ 0.5 s). In the Gustatory variant, there are no auditory cues, whereas, in the Start task, the Start cue (purple ticks) signals that a new tastant becomes available. In the Start/Stop task there is also a Stop (white noise) cue that commences at RE and continues until the timeout of the Stop licking epoch has finished. **(C–E)** Representative raster plots from three different animals (upper panels) and PSTHs (bottom panels; all aligned to RS, time = 0 s) of licking responses (red ticks) illustrating the Gustatory **(C)**, the Start **(D)**, and the Start/Stop **(E)** variants of the brief-access tests. In all cases, trials were sorted as a function of the sucrose concentration (see colored scale at right) and lick bout duration. The horizontal blue lines separate Complete from Incomplete trials. The Inset (dashed rectangle in **D**) is a zoom from the raster plot of the Incomplete trials in a Start brief-access test, highlighting a large number of trials with one-lick bouts (see arrow). The upper PSTHs was for all (Total) trials, the middle panels for Complete trials, and the bottom panels for Incomplete trials. Note that the licks/s in the Incomplete trials track the sucrose palatability.

Figure 1A shows a schematic representation of the behavioral set-up. All experiments were performed in a sound-attenuating cubicle in an operant box (Med Associates, St. Albans, VT). The box contained a central V-shaped licking port equipped with a photo-beam sensor (Med Associates, St. Albans, VT) to register individual licks. In this port, a single midline spout delivered, in every lick during the Reward epoch, 10-μL of tastants directly onto the rats' tongue. The spout, always in a fixed position, was available at all times and consisted of 5 independent 20-gauge stainless-steel needles cemented together with dental acrylic from which a different tastant was delivered (Gutierrez et al., 2010). Each blunted needle was connected to a solenoid valve and maintained under constant air pressure to ensure precise delivery of liquid (in a 0.01 s pulse width; calibrated before each session). To control for any differences in the valve's sounds, every day a different valve released a different tastant. In addition, the box contained a speaker that generated a white noise and a “click”-sound generator (Med Associates).

We designed three variants of the one-sipper brief-access test: A Gustatory test and two gustatory plus two auditory stimuli tasks, named Start and Start/Stop. The three tasks had a similar trial structure and were composed of the following epochs: Initiation, Reward, Empty licks and Stop licking. In the Gustatory task, for a given trial the subject had to first lick the sipper to initiate the 5 s Reward epoch during which every lick was rewarded with a randomly selected sucrose solution or water (from 0 to 20 wt%). Thus, the “Trial Type” variable in the subsequent discussion refers to the delivery of various sucrose concentrations and water. The start and end of the Reward epoch were denoted Reward Start (RS) and Reward End (RE), respectively. After the RE, the Empty licks epoch began. Tastants were not delivered in this epoch. To initiate a new trial, animals were required to refrain from licking the empty sipper for a variable period of 1–3 s (Stop licking epoch). Within the Reward epoch, for any sucrose concentration or water, the subjects were free to either produce a single licking bout (licks with Inter-Lick Intervals [ILIs] < 0.5 s) or emit two or more licking bouts (separated by ILIs ≥ 0.5 s; Smith et al., 1992). Trials with one bout that spanned the entire 5 s Reward epoch were designated “Complete,” whereas trials with more than one lick bout were designated, “Incomplete.” Lick bouts can be composed of one or more licks. A one-lick bout is when the animals licked just once, and that lick was followed by an ILI ≥ 0.5 s.

To evaluate the impact that external auditory cues have upon sucrose's palatability, we designed two additional variants of the Gustatory brief-access taste test by combining gustatory stimuli with auditory cues. In these tasks, except for the addition of auditory stimuli, the conditions were identical to those used in the Gustatory test. In the Start task an auditory click indicated the beginning of a new trial (at the Initiation epoch onset). In the Start/Stop task, the same start click was presented, but also, at every RE a white noise was turned on indicating the termination of the Reward epoch. When a subject stopped licking for at least 1–3 s of the inter-trial interval the auditory stop signal was turned off. It is important to note that the Stop cue was not a threatening auditory stimulus (O'Connor et al., 2015) since initially it does not interrupt the animals licking (data not shown). Therefore, to refrain from licking rats learned to use the Stop cue.

We operationally defined sucrose's palatability as the enhancement of some hedonically positive oromotor responses triggered by increasing concentrations of sucrose (Berridge and Grill, 1983; Spector et al., 1998). Specifically, oromotor responses include either an increase in the lick rate or bout size (e.g., first bout duration). For each brief-access test, we analyzed palatability oromotor responses as follows: Peri-Stimulus Time Histograms (PSTHs) of licking were generated as a function of the sucrose concentrations delivered in each trial (hereafter called Trial Type) and the responses were aligned (time = 0 s) to the RS. We also obtained the PSTHs of licking as a function of Complete and Incomplete trials. The empirical cumulative distribution functions [CDFs, using the Kaplan-Meier method (Kaplan and Meier, 1958)] of the lick rates were constructed during the Reward epoch as a function of Trial Types. A similar analysis was performed for the distribution of the first lick bout duration. The lick rates, lick bout durations, and other behavioral distributions were described by their medians and interquartile ranges (IQRs).

To account for differences in behavioral strategies used by the animals to solve each brief-access test, in every training session we calculated the Bout index- defined as the number of Complete trials over the number of both Complete and Incomplete trials. Additionally, as a measure of intake dynamics for each task, we calculated the cumulative sum of rewarded licks throughout the sessions. Caloric intake was calculated by adding the number of licks given to each sucrose concentration during the Reward epoch. To determine the time needed to identify the end of the reward delivery, we calculated for the Complete trials the latency to stop empty licking after the RE.

To further confirm the impact that exteroceptive auditory cues have upon sucrose's palatability, we performed a licking microstructure analysis. Following Davis and Smith (1992) we calculated the number of lick bouts and their duration. For this analysis, we took all the lick bouts in a trial (determined as trains of contiguous licks with an ILI < 0.5 s) using only the rewarded licks during the Reward epoch (**Table 2**). The lick bout duration was defined as the time elapsed between the first and last lick in a bout.

### Electrophysiology

While the subjects were performing one of the behavioral tests, extracellular single-unit activity from the NAcSh was recorded using a Multichannel Acquisition Processor (Plexon, Dallas, TX). Specifically, voltage signals were sampled at 40 kHz, and digitalized at 12 bits resolution. Single unit timestamps were extracted from the raw signal, by using an online band-pass filter with a low cutoff 154 Hz and high cutoff 8.8 KHz. Only single neurons with action potentials having greater than 3:1 signal to noise ratio were analyzed (for an example, see Figure S2A). The action potentials were isolated on-line using voltage-time threshold windows and a three principal components contour template algorithm. Furthermore, for all recordings, we performed off-line spike sorting (Plexon offline sorter), and only single units with stable waveforms across the entire session were included in the analysis (Gutierrez et al., 2010) (see Figure S2B). Neurons recorded in all sessions were collapsed and included in the analysis. We acknowledge that it is possible to observe, a few examples, where the same neuron was putatively recorded across several sessions that is their waveforms, inter-spike-interval distributions, and type of modulation during the task were similar across days. See Figures S2C,D for an example of a Palatability-related neuron. Nevertheless, in this study we did not attempt to identify whether or not an electrode recorded the same neuron across different sessions (Lütcke et al., 2013) and thus the neurons were regarded as independent.

To identify neurons with either palatability (oromotor responsiveness) related information or with an activity that was modulated with increasing concentrations of sucrose during the three brief-access tests, we developed two analyses from NAcSh spikes that separate oromotor and sucrose concentration-dependent components of feeding.

#### Oromotor palatability-related neurons

To identify neurons whose firing rate correlated with oromotor type palatability, for each recording session a Palatability index was developed. This index was designed to reflect the overall envelope of the rats' oromotor responses elicited by each sucrose concentration. It was computed by averaging the lick rate (number of licks/5 s) during the entire Reward epoch, including all the trials (i.e., Total = Complete and Incomplete trials). Since consummatory licking is a stereotypic behavior in rats occurring at a frequency of ~7 Hz (Davis, 1973), the Palatability index can take physiologically relevant values between 0–7 Hz for each concentration. That is, 0 Hz means that in all trials the animals completely rejected a particular solution, whereas 7 Hz indicates that, in all trials, the animals licked continuously during the entire Reward epoch, thus reflecting more palatable responses elicited by the tastant. Then, the firing rate of each neuron was calculated for a variety of time centers (from 0.25 to 4.75 s in steps of 0.5 s, relative to RS) and in multiple window sizes (from 0.5 to 5 s in steps of 0.5 s), such that each window was estimated as the center ± (window size/2). Thus, using the previous parameters in the Reward epoch a total 33 different time windows were evaluated. Hence, we identified the windows where the mean firing rate was significantly different for at least one Trial Type (i.e., concentrations; using a Kruskal-Wallis test at an alpha of 0.05). Next, on a trial-by-trial basis, we computed the Pearson's correlation coefficient (r; the alpha level at 0.05) between both the Palatability index and the firing rate. The window with the largest absolute Pearson's correlation coefficient was selected as the “best-window.” Thus, for the “best-window” and each statistical test (i.e., Kruskal-Wallis test and Pearson correlation) a permutation assay was used for multiple-testing correction (Davison and Hinkley, 1997). This analysis was accomplished by shuffling the Trial Types labels 20,000 times (using the Matlab function “shuffle”). A corrected *p*-value was obtained using the following formula *p* = (k+1)/(n+1), where k is the times in which a permuted distribution leads to a *p*-value smaller than the original *p*-value and n is the number of repetitions (North et al., 2002). Only time-windows with *p'*s < 0.05 in both tests (Kruskal-Wallis and Pearson correlation) were considered as statistically significant (note that if a neuronal response was significant in two independent statistical tests, then the final alpha level was of 0.05^*^0.05 = 0.0025). Thus, the “best-window” is the one where the firing rate maximally correlated with the oromotor responses elicited by sucrose's palatability. Similar results were found if the Palatability index was computed using the bout duration instead of the lick rates (data not shown). This is because, in the Reward epoch, the lick rate and bout duration were highly correlated (all *r*'s = 0.9; all *p*'s < 0.001).

#### Sucrose concentration-dependent neurons that do not change with licking rate

To determine whether in the absence of differences in licking responses (Complete trials), NAcSh neurons could discriminate sucrose in a concentration-dependent manner, we repeated the same analysis described above but now for Complete trials, and using the sucrose concentration as an independent variable for the linear regression. We then calculated the mean firing rate during every possible time-window generated throughout the Reward epoch. This process guarantees that changes in neuronal activity are driven by monotonic increases (or decreases) in sucrose concentration and not by differences in licking behavior. The same permutation test, described above, was used here to correct for multiple-testing and every neuronal response that exhibited in any time-window both statistically significant differences in the Kruskal-Wallis test (α = 0.05) and a significant Pearson correlation (α = 0.05) were considered to be sucrose concentration-related neurons. Again, the time-window with the highest Pearson's r-value was selected for each neuron. Neurons that correlated with both the Palatability index and sucrose concentration were assigned to the category which they had the greatest Pearson's coefficient.

#### Licking coherent-sucrose dependent neurons

A multitaper spectral analysis was used to calculate the coherence between licking and spiking activity during the Reward epoch by segmenting the PSTHs of these events into chunks (Jarvis and Mitra, 2001). The coherence (C) between licking and the spike trains was computed using the following formula:

$$\begin{array}{l} C\left(f\right)=\;\frac{I_{xy}}{I_{xx}I_{yy}} \end{array}$$

Where Ixx represents the spectrum of licking behavior, *Iyy* is the spectral analysis of neuronal activity, and *Ixy* is the cross-spectrum of licking and spike spectrum. Note that the coherence (C) is normalized to range between 0 and 1. Finally, *f* is the frequency where coherence was computed (4–7 Hz, which corresponds to the bandwidth of licking frequency). The confidence interval, *C(f)*, and significance threshold (with an alpha of 0.05%) were computed with a jackknife method with a finite size correction, using the procedures developed by (Jarvis and Mitra, 2001). A neuron was classified as licking-coherent only if its lower confidence interval (95%) crossed the significance threshold.

The trial-by-trial coherence values of licking-coherent neurons during the Reward epoch (C were tested using a Kruskal-Wallis test). Those with significant differences in at least one Trial Type were named “coherent taste”-coding neurons. Then, we calculated the Pearson's correlation coefficient (r) between the coherence vs. Trial Types. Coherent taste-coding neurons that exhibited significant correlations were classified as either Increased or Decreased as a function of their Pearson's r sign. The Kruskal-Wallis and Pearson's correlation tests were corrected by a permutation test with α = 0.05 as described above.

#### Licking active and inactive neurons

Following Krause et al. (2010), NAcSh neurons were characterized according to their typical modulation during licking. This analysis was accomplished using a one-way ANOVA to compare firing rates during a baseline (−1.5 to −0.5 s) period, against the activity in the Reward epoch (0–5 s aligned to RS). To prevent contamination from the anticipatory activity in the Reward epoch, we avoided using times immediately before the onset of licking (−0.5 to 0 s). All Trial Types were combined to identify the overall modulation caused by licking regardless of the sucrose concentration. Neurons that significantly increased their firing rate after the RS were named “Lick-Active” and neurons that decreased their activity were named, “Lick-Inactive.” Neurons with no significant modulations were named, “Not modulated” (Tellez et al., 2012).

#### Phasic activations/inhibitions at auditory cues

To identify neurons carrying information about the auditory Start and Stop cues we used a cumsum statistics (Gutierrez et al., 2006). This procedure identified for which time-bins (0.05 s resolution) the spiking activity significantly increased relative to baseline from −3 to 0 s before the onset of an auditory cue. Since auditory cues were not given in the Gustatory test, and no Stop cue was given in the Start test, the PSTHs were aligned to a time point where these events theoretically should have occurred. Although some neurons exhibited biphasic modulations, to classify the neuronal response we used the sign of the first significant modulation from the baseline.

To test at the RE whether phasic inhibitions could be evoked, *cumsum* statistics were used to search for neuronal activity from 0 to 1.5 s relative to the RE that significantly decreased compared with two baselines. The first baseline spanned from −1.5 to −0.5 s before the RS and the second before the RE (from −2 to 0 s). Neurons that met these two criteria were classified as phasically inhibited. This approach guarantees that inhibitions that occurred after the RE were significantly below baseline activity. To avoid including tonic modulations, significant decreases that continued for more than 1.5 s after the RE were removed and only the first significant modulation was used in subsequent analysis.

#### Population decoding of sucrose

A NAcSh population decoding of sucrose's concentration/palatability attributes was also tested for the three brief-access tests. This analysis was accomplished by using a population decoding algorithm (Matlab toolbox of the 1.0 version of the Neural Decoding Toolbox, www.readout.info) (Meyers, 2013) to classify trials as a function of the sucrose concentration. All neurons recorded in different sessions were pooled together as if they have been recorded at the same time. Additionally, to determine the quantity of information contained in the licking responses about the palatability/concentration of sucrose, we performed the same population decoding algorithm, but now using only the PSTHs of the lick rate. These analyses were done by first assigning neurons to a different neuronal population as follows: Palatability-related, Concentration-related, Coherent, Lick-Active, Lick-Inactive, Modulated (pooling all the latter neurons in one group), and Not modulated (the ones that did not belong to any of the groups mentioned above). Note that a neuron can belong to more than one group. Then, the PSTHs of the activity within each population and the PSTHs of the licking responses were computed (0.05 s bins, aligned to the RS), normalized to Z-scores (by using the zscore_function to normalize_FP function in Matlab) and averaged as a function of each class (Trial Types). A classifier (max_correlation_coefficient_CL classifier function) was trained with these mean population vectors per class from a training set of trials (templates) and tested by calculating the Pearson's r coefficient between the templates and the testing trials set. The classes that yielded the highest correlation per trial were used by the classifier as decision values to predict the Trial Type. To increase statistical power, this classification was repeated three times, in each of them, a cross-validation procedure was performed by splitting the data into k splits (being k the number of trials). The classifier was then trained using k-1 data sets, and it was tested in the remaining trial. This procedure was repeated until all the trials were tested. The number of k splits was determined by the minimal number of trials that occurred in each class (Trial Types) within a population. We ran the algorithm with a different number of k splits (from 10 to 17 trials, for each Trial Type) and the performance in every time bin was tested with a one group right-tailed t-test to know if they were significantly different from the chance level (20%). To determine the relative contribution of each neuronal population in the overall decoding of the Modulated neurons we performed, for each behavioral test, a one-population drop analysis. For this analysis, we repeated the calculation of the decoding performance during the Reward epoch but dropped one population at a time (i.e., Palatability, Concentration, Coherent, Active, or Inactive). The decoding performances of these new populations and all the Modulated neurons together were compared using Kruskal-Wallis tests. As a control procedure to account for the impact of reducing the member's number of the Modulated population, we performed random-drop decoding based on the size (n) of the population that most impaired the performance (best population). This analysis consisted of dropping a randomly-selected population of neurons (of size n) from the Modulated group. We then calculated the decoding performance 100 times but using a new random population each time and average them to compare their mean performance against the best decoding population (by using a Kruskal-Wallis).

All data processing and statistical analysis were performed using MATLAB (The MathWorks Inc., Natick, MA) and StatView 4.57 (Abacus Concepts, Inc., Berkeley, CA). Unless otherwise mentioned, for all statistical tests, the alpha level was set at 0.05. In Supplementary Methods can be found the complete statistical results of all the figures in the manuscript.

## Results

### Behavioral results

Details of the three variants of a brief-access test (i.e., Gustatory, Start, and Start/Stop) are seen in Figure 1. One goal of these tests was to determine how exteroceptive auditory cues could modulate the palatability responses elicited by sucrose under conditions where the animals themselves decide when to initiate and stop licking. Another goal was to determine the effects of reducing the temporal uncertainty by the auditory cues that signal when the Reward epoch begins and ends. In these three tests if animals licked continuously (ILIs < 0.5 s) in the 5 s Reward epoch, trials were classified as Complete, whereas trials were classified as Incomplete if during the Reward epoch animals paused licking for ≥ 0.5 s.

**Figure 2:**
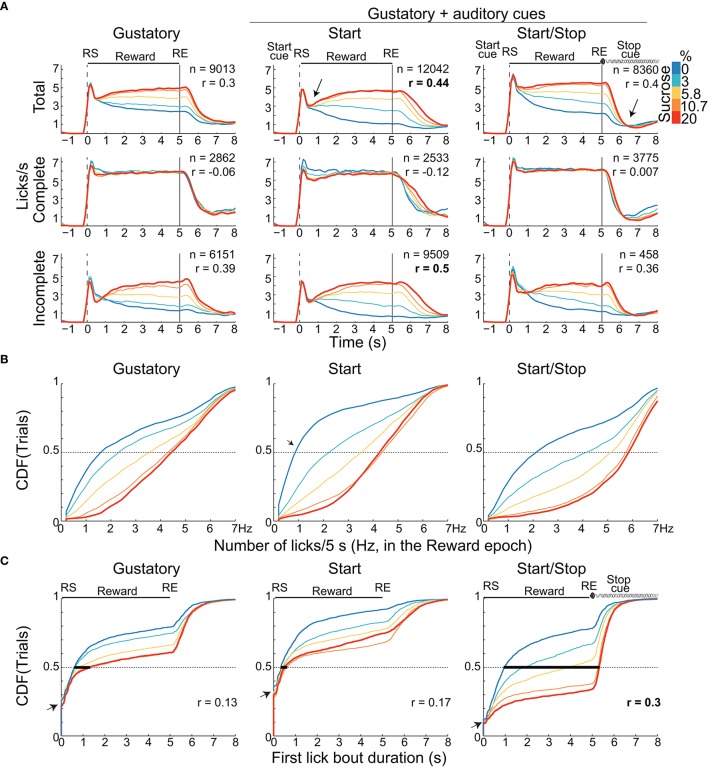
Animals exhibit distinct palatability (behavioral) responses that depend on the presence of auditory cue(s). **(A)** The lick rates (licks/s) for the three brief-access tests aligned to the RS (vertical dashed line) as a function of sucrose concentration. The licking responses are shown for the “Total” trials (upper), for Complete (middle) and Incomplete trials (bottom panels). The n's are the number of trials pooling across animals and sessions. Only the Total and Incomplete trials significantly discriminate among the sucrose concentrations. The arrow in the PSTHs of the Start test highlights the abrupt decrease in lick rate for all tastants after which the lick rate tracks the sucrose concentration. The arrow just after RE in the Start/Stop test indicates that animals rapidly stopped licking. Also shown is the Pearson's correlation (r's) between the lick rate in the Reward epoch and the sucrose concentrations. **(B)** Cumulative distribution functions (CDFs) for the lick rate (total number of licks during the entire 5 s Reward epoch). **(C)** The CDFs of the first bout duration over the range of RS for the three tests. The bold horizontal lines at 0.5 on the Y-axis indicate the median bout duration for each sucrose concentration. Note that the first lick bout duration was largest in the Start/Stop test and it was the most positively correlated with sucrose concentrations (see r's). The small arrows in the CDF's indicate the fraction of trials where animals made their first lick bout with a one-lick (bout durations equal 0 s).

We initially characterized how auditory cues modulate positive hedonic oromotor responses elicited by sucrose's palatability. Figures 1C–E display, for each brief-access test, representative raster plots from three rats. It is seen that the number of Incomplete trials for water (0% sucrose) was greater than those for sucrose. Also, we note that the lick rate for the Complete trials does not correlate with the sucrose concentrations. In contrast, for the Incomplete trials, the lick rate tracks the sucrose concentration, with the higher the concentration, the greater the palatability or lick rate. The same behavior is seen for the population responses (Figure 2A). That is, the population PSTH shows that in the Complete trials there is no correlation of the lick rate with the sucrose concentration [middle panels; Pearson's *r* = −0.06 (Gustatory); *r* = −0.12 (Start); and *r* = 0.007 (Start/Stop)]. In contrast, for the Incomplete trials, the lick rate linearly increased with the sucrose concentration [Figure 2A bottom panels: *r* = 0.39 (Gustatory), *r* = 0.5 (Start), and *r* = 0.36 (Start/Stop) all *p*'s < 0.001]. Also, note that the lick rate correlates better with palatability in the Start test than for the other two tests.

We next explored how rats licked during the start and end of the Reward epoch. As seen in Figure 1D, in the raster plot for the Incomplete trials in the Start test, a rat that initially sampled a tastant with one lick (61% of trials; see expanded area around RS and red ticks at time = 0 s, see arrow inset in the Figure 1D dashed rectangle) and after a variable pause (>0.5 s) it re-engages in a second longer bout that is clearly seen for the trials with 20% sucrose. The same behavior was observed in the population responses (Figure 2A). That is, the initial lick rate was always highest at RS (time = 0 s), followed by a pause, which was accompanied afterward by a concentration-dependent increase in the lick rate. Of the three tasks, the Start test showed the greatest initial decrease in lick rate (see arrow Figure 2A middle panel). Another difference between the tasks, seen in Figure 2A, is that the animals stopped licking faster in the Start/Stop task than in the other two tasks [see arrow in Figure 2A Start/Stop; the medians and (IQR's) were: Gustatory: 0.9 s (1.06); Start: 1.12 s (0.98) and Start/Stop: 0.58 s (0.63)]. Thus, animals learned from the Stop cue to stop licking faster a result that will be discussed in more detail below.

#### In the start test sucrose's palatability is enhanced by increasing the lick rate relative to water

To determine whether the lick rate during the 5 s Reward epoch reflected sucrose's palatability, we counted all the licks in this epoch as a function of Trial Type and constructed CDFs (Figure 2B and Table 1 for means ± sem). In all tasks, the lick rate increased as a function of sucrose concentration [two-way ANOVA; *F*_(2, 4)_ = 1325, *p* < 0.0001]. The lick rates were in the order: Start/Stop > Gustatory > Start. The relatively low lick rates in the Start test can be rationalized by noting that the CDF was skewed, such that the medians for 0% sucrose (water) were 0.8 licks/ 5 s (see arrow Figure 2B), whereas it was 1.6 licks/5 s for the Gustatory test (see Figure 2B). This result suggests that the Start cue facilitated the rejection of water, but without increasing the lick rate for high sucrose concentrations (the medians were 4.3 and 4.4 licks/5 s for 20% sucrose; Start test vs. Gustatory, respectively). By normalizing the sucrose lick rate relative to water trials (see Table 1 for the Tastant/Water ratios), it is seen that the Start test was the task that most enhanced sucrose palatability relative to water.

**Table 1:** The lick rate (licks/s) for the three brief-access taste tests as a function of sucrose concentration.

**Sucrose (wt%)**					
	0	3	5.8	10.7	20
**Total trials**					
Gustatory	2.65 ± 0.05	3.1 ± 0.05	3.7 ± 0.05	4.24 ± 0.04	4.44 ± 0.04
Start	**1.73 ± 0.04**	**2.85 ± 0.04**	**3.53 ± 0.04**	4.22 ± 0.04	**4.23 ± 0.03**
Start/Stop	**3.01 ± 0.06**	**3.94 ± 0.06**	**4.63 ± 0.05**	**5.35 ± 0.04**	**5.56 ± 0.04**
**Complete trials**					
Gustatory	6.07 ± 0.05	3.21 ± 0.04	6 ± 0.04	5.87 ± 0.04	6 ± 0.04
Start	6.14 ± 0.05	**5.92 ± 0.04**	**5.72 ± 0.04**	5.79 ± 0.03	**5.72 ± 0.03**
Start/Stop	**6.44 ± 0.04**	**6.39 ± 0.03**	**6.32 ± 0.03**	**6.35 ± 0.03**	**6.43 ± 0.03**
**Incomplete trials**					
Gustatory	1.75 ± 0.04	2.04 ± 0.04	2.51 ± 0.04	3.17 ± 0.04	3.43 ± 0.04
Start	**1.35 ± 0.03**	**2.22 ± 0.03**	**2.9 ± 0.04**	**3.48 ± 0.04**	**3.73 ± 0.03**
Start/Stop	**2.07 ± 0.04**	**2.75 ± 0.05**	**3.29 ± 0.06**	**3.75 ± 0.07**	**3.92 ± 0.07**
**Tastant/water ratio**					
	0	3	5.8	10.7	20
**Total trials**					
Gustatory	1 ± 0	1.19 ± 0.03	1.47 ± 0.04	1.74 ± 0.07	1.83 ± 0.08
Start	1 ± 0	1.8 ± 0.06^*^	2.32 ± 0.1^*^	2.8 ± 0.15^*^	2.8 ± 0.13^*^
Start/Stop	1 ± 0	**1.44 ± 0.04**	**1.71 ± 0.05**	1.99 ± 0.08	2.07 ± 0.08

*Values are (mean ± sem). Data in bold means p < 0.05, in comparison with Gustatory task. All lick rates (licks/s) were statistically different relative to water (0%) at an alpha level of 0.05. In the Tastant/Water ratio data*,

* *means p < 0.05, in comparison with the Start/Stop task*.

#### The start/stop test enhances sucrose's palatability by increasing the first bout duration

To elucidate how the Start/Stop cues improved sucrose's palatability, we calculated the first lick bout duration as a function of Trial Type (Figure 2C). We found that the first lick bout duration was largest for the Start/Stop test followed by the Gustatory and then the Start test [two-way ANOVA; *F*_(2, 4)_ = 686.5; all *p*'s < 0.001]. This result indicates that the Start/Stop cues also enhance palatability responses by increasing the first bout duration as a function of sucrose concentration [*F*_(2, 4)_ = 316.4, *p* < 0.0001].

To further confirm that exteroceptive auditory cues enhanced oromotor responses elicited by sucrose, we quantified the number and duration of lick bouts in the Reward epoch. For the three tests, the licking microstructure analysis revealed that the bout duration increased as a function of sucrose concentration, thereby validating the use of the brief-access tests to measure palatability (Young and Trafton, 1964; Davis, 1973; Smith et al., 1992). Importantly, we found that relative to the Gustatory test, for the two higher sucrose concentrations tested the Start test exhibited a larger bout duration, and the Start/Stop test showed a fewer number of bouts, but having bouts of larger duration in comparison to both the Start and Gustatory tests (Table 2). As previously shown (Spector et al., 1998), the smaller the number of bouts and greater their duration the more palatable is a tastant. In sum, this finding confirmed that auditory cues enhanced sucrose palatability and that as measured by the average bout duration (see Table 2), the Start/Stop test elicited the largest enhancement,.

**Table 2:** Analysis of the licking microstructure revealed that exteroceptive auditory cues enhanced oromotor licking responses elicited by sucrose's palatability.

**Sucrose (wt%)**	**Gustatory**		**Start**		**Start/stop**	
	**Number of bouts**	**Bout duration (s)**	**Number of bouts**	**Bout duration (s)**	**Number of bouts**	**Bout duration (s)**
0	2 ± 0.03	1.2 ± 0.03	**1.8 ± 0.02**	**0.8 ± 0.02**	**1.5 ± 0.02**	**1.6 ± 0.04**
3	2.2 ± 0.03^*^	1.3 ± 0.03	2.2 ± 0.02^*^	1.2 ± 0.03^*^	1.6 ± 0.02^*^	2.1 ± 0.04^*^
5.8	2.4 ± 0.04^*^	1.5 ± 0.03^*^	2.4 ± 0.03^*^	1.6 ± 0.03^*^	1.6 ± 0.02^*^	2.6 ± 0.04^*^
10.7	2.3 ± 0.04^*^	1.9 ± 0.04^*^	2.1 ± 0.02^*^	2.1 ± 0.03^*^	**1.5 ± 0.02**	3.2 ± 0.05^*^
20	2.3 ± 0.03^*^	2 ± 0.04^*^	2.3 ± 0.02^*^	2.1 ± 0.03^*^	**1.5 ± 0.02**	3.3 ± 0.05^*^

*Values are mean (± sem). Data in bold means statistically different against the Gustatory task*.

* *Indicates statistically different relative to water (0%). The alpha level was set at 0.05*.

#### In the start test, rats tend to initially sample a tastant with one single lick

Another observation that can be seen in Figure 2C is that a large component of the distribution in the Gustatory task was composed of trials with bouts of 0 s duration (left panel, y-axis at 0.25, see arrow), meaning that in 25% of the trials, the first bout contained only a single lick. This behavior was more commonly found in the Start test, since 31.5% of trials were of one-lick in the first bout (also see inset Figure 1D) than in the Start/Stop task, which had one lick bouts only 11% of the sample (Figure 2C arrow right panel; χ^2^ = 8.16, *p* < 0.01, for Start vs. Start/Stop). In this regard, and unlike in the Start/Stop test, the Start cue alone promotes the behavior of sampling a tastant with one lick and then, after a pause, begins a second larger licking bout resulting in a fragmented licking pattern. In sum, these data indicate that rats use different behavioral strategies that depend on the auditory cues context to express sucrose palatability responses (see Table 2).

#### Rats in the start/stop test have more complete trials and consume the most calories

**Figure 3:**
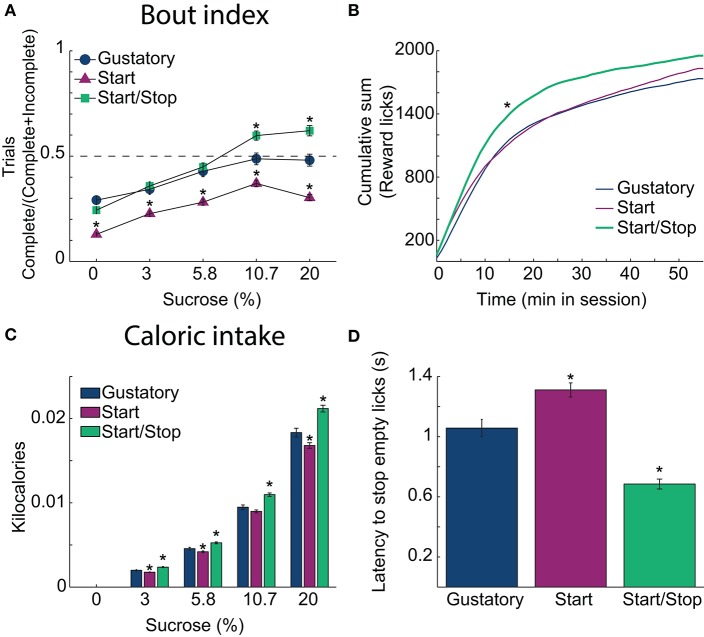
Auditory Start/Stop cues increase the Bout index, number of rewards obtained and caloric intake. **(A)** The Bout indices (the ratio of the number of Complete trials to the number of Complete and Incomplete trials) for the three brief-access tests plotted as a function of sucrose concentration. The order was: Start/Stop (green) > Gustatory (blue) > Start (purple). Values are mean ± sem across trials. **(B)** The cumulative sum of rewarded (wet) licks in the Reward epoch over the 55 min session for each test. The Start/Stop test had the largest number of rewarded licks. **(C)** A histogram is displaying the average caloric intake as a function of sucrose concentration for the three brief-access tests. Animals tested in the Start/Stop task consumed significantly more kilocalories (sucrose) than those in the other two brief-access tests. *N* = 91 sessions for the Gustatory task; 109 for the Start task; 97 for the Start/Stop task. **(D)** The latency to stop licking the empty sipper after the end of the Reward epoch (RE) for each test. ^*^Indicates significant differences with an alpha level of 0.05 in comparison with the Gustatory task in all panels.

In each brief-access test (Figure 3), we also further explored how animals lick during the Reward epoch. This was achieved by calculating the Bout Index (Figure 3A; Bout Index = #Complete/(#Complete + #Incomplete trials). This index reflects the extent that animals will perform a Complete 5 s trial. Compared to the Gustatory test, the Bout Index for the Start/Stop task was larger and the Start test smaller (all *p*'s < 0.001). Interestingly, the Bout Index linearly increased from water until 10.7% sucrose (all *p*'s < 0.0001), where it plateaued (*p* = 0.28 n.s. between 10.7 and 20%). Thus, in the Start/Stop test, where animals are more certain about the beginning and end of the Reward epoch, they feed more continuously.

To ascertain how the behavioral context affects caloric intake, we calculated the number of sucrose rewards consumed. Figure 3B shows in each brief-access test the cumulative sum of (wet) licks in the Reward epoch. Rats in the Start/Stop test obtained more liquid rewards and obtained them more rapidly than in the other tests, suggesting an increase in motivation (Kolmogorov-Smirnov test; for Gustatory D = 0.25; *p* = 0.05 and for Start test, D = 0.41; *p* < 0.001). Accordingly, the effect on the final caloric intake (Figure 3C) was significantly different among tests [two-way ANOVA; *F*_(2, 3)_ = 72, *p* < 0.0001] and sucrose concentrations [*F*_(2, 3)_ = 2760.3, *p* < 0.0001, with a significant interaction *F*_(6, 1176)_ = 12.6, *p* < 0.0001]. Specifically, rats in the Start/Stop test consumed more calories in comparison with the other two tests (all *p*'s < 0.001), whereas animals in the Start test consumed the fewest calories (all *p*'s < 0.001). Thus, the caloric intake is dependent on the behavioral context in the form of auditory stimuli.

#### Rats stopped licking an empty sipper fastest in the start/stop test

To measure how the auditory context affects feeding, we calculated the latency to stop licking an empty sipper. Specifically, relative to the Gustatory test, rats stopped licking faster in the Start/Stop test, whereas they were slower in the Start test. Figure 3D shows that differences among tasks were significant [one-way ANOVA; *F*_(2, 291)_ = 46.5, *p* < 0.0001]. In comparison to the Gustatory test (no auditory cues), the Start test had a longer latency (*p* = 0.002) and the Start/Stop test had the shortest latency (*p* < 0.001 vs. Gustatory test; also see arrow in Figure 2A, upper right panel). Thus, rats used the Stop cue to refrain from licking more rapidly. In summary, these behavioral studies show that exteroceptive auditory cues can modify sucrose palatability by changing the animals licking patterns.

### Electrophysiological studies in the NAcSh related to behavior

**Figure 4:**
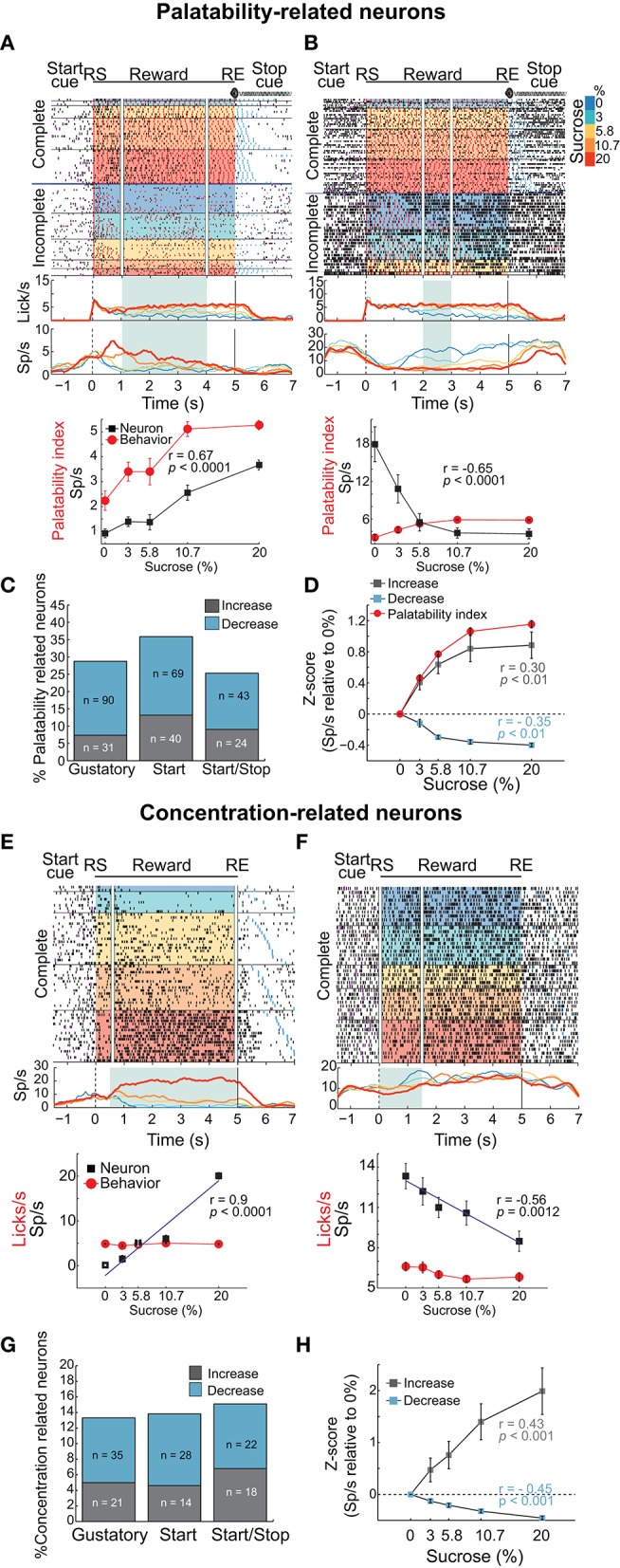
Distinct neuronal populations in the NAcSh track either sucrose evoked palatability oromotor responses or the sucrose concentration. **(A)** A raster plot showing a single unit NAcSh response obtained during a Start/Stop test. The action potentials, depicted as black ticks, are shown as a function of the color-coded sucrose concentrations for Complete and Incomplete trials. Below are the PSTHs of the lick rate and the neuronal activity (Sp/s) for all trials that are aligned to the start of the reward epoch (RS). The vertical white lines in the raster plot and the green rectangle on PSTH depict the time-window where occurs the maximum correlation (Pearson's r) between neuronal activity and Palatability index (i.e., the average lick rate as a function of Trial Type for this session). The bottom panel shows the Palatability index (red) and the number of spikes (black) over the analyzed window with a Pearson correlation (*r* = 0.67, *p* < 0.0001). **(B)** A representative example of a neuronal response with a negative correlation with the Palatability index (r = −0.65, *p* < 0.0001). Conventions are same as above. Note that briefly before and during the first second of the Reward epoch that this neuronal response was inhibited in a manner independent of the sucrose concentration. **(C)** A histogram showing the percentage of neurons in each task that significantly correlated with sucrose palatability oromotor responses. The plot shows neurons with negative (*r* < 0, blue) or positive correlations (r > 0, gray) with sucrose palatability. The n's referring to the actual number of neurons in each subset. Gustatory vs. Start, *p* = 0.14; Gustatory vs. Start/Stop, *p* = 0.45; and Start vs. Start/Stop, *p* = 0.047 **(D)** Mean ± sem of normalized activity (in Z-score relative to activity in water trials) for all neurons with either positive or negative correlation with the Palatability index (r = 0.3 and r = −0.35, respectively; *p*'s < 0.01). Also shown is the Palatability index (red line, also normalized to water lick rate) and its corresponding correlation coefficients with NAcSh neurons. **(E)** A raster plot of neuronal responses recorded in a Start test constructed only with Complete trials. In these trials, the licking rate is independent of the sucrose concentration. For visualization purposes, except the last lick in the empty lick epoch (see blue ticks), the ticks for licks were omitted. Vertical white lines in the raster plot and the green rectangle in the PSTH depicts the time-window where the Pearson correlation between neuronal activity and sucrose concentration was maximal. The bottom panel shows the average (± sem) of the neuronal activity during the time-window that maximizes the correlation with the sucrose concentration (*r* = 0.9, *p* < 0.001). Also shown is the sucrose-independent lick rate in the same time-window (red line). **(F)** Representative example of a neuronal response whose firing rate was anti-correlated with the sucrose concentration (*r* = −0.56, *p* = 0.0012). It is seen that within the first 1.5 s this neuron fired more for 0% (water) than for 20% sucrose. **(G)** Histogram showing the percentage of neurons in the Complete trials from the three brief-access tests that significantly correlated with the sucrose concentrations. The n's indicate the number of neurons in each category. Gustatory vs. Start, *p* = 0.86; Gustatory vs. Start/Stop, *p* = 0.57; and Start vs. Start/Stop, *p* = 0.7 **(H)** The average (± sem) activity of all neurons with sucrose concentration related information that either increase or decrease their activity as the sucrose concentration increased. The average Pearson's r coefficients are also shown for Increases and Decreases (*r* = 0.43 and *r* = −0.45, respectively; *p*'s < 0.001).

**Figure 5:**
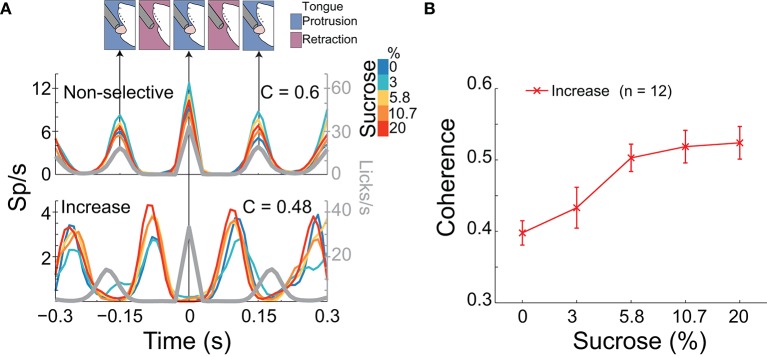
A population of NAcSh neurons can track sucrose concentrations by increasing their coherence with licking. **(A)** Upper: A schematic of the temporal sequence of a rat licking a sipper (time = 0 s) and PSTHs of the activity of two lick-coherent neurons aligned to all licks in the 5 s Reward epoch. The upper example is from a neuronal response that was recorded in a Gustatory test whose coherence was similar across Trial Types (see color bar inset; *r* = 0.03, *p* > 0.05) and that fired when the animal's tongue made contact with the sipper. Lower: This response shows a lick-coherent neuronal response (recorded in a Start/Stop task) that increased its coherence in a sucrose-concentration dependent manner (*r* = 0.45, *p* < 0.05) and that was phased locked to tongue retraction. The gray trace indicates the lick rate (and time between arrows indicates a lick cycle that is each time the animal's tongue make contact with the sipper). **(B)** The mean (± sem) of the coherence of all 12 neurons that significantly increased their synchrony with licking as a function of sucrose concentration.

**Figure 6:**
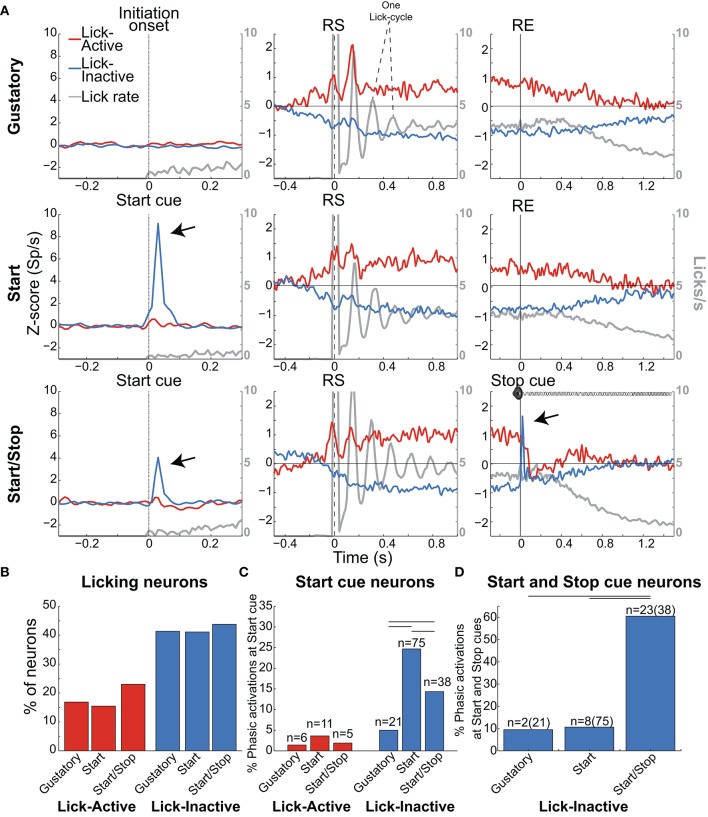
Lick-Inactive NAcSh neurons respond phasically to both the Start and Stop auditory cues. **(A)** The population activity of neurons (Z-score) that was either Active (red) or Inactive (blue) for times around the initiation of licking and during and after the Reward epoch for the Gustatory (upper), Start (middle) and Start/Stop test (bottom panels). Responses to all (Total) licks (licks/s) are shown in gray and the time between peaks indicates one lick cycle (~150 ms) and aligned to the onset of the Initiation epoch (for the Gustatory test) and the Start cue for the Start and Start/Stop tests (left column). For each brief-access test neuronal responses were also realigned to the reward start (RS -middle column) and the reward end (RE (right column). The arrows shown in the Lick-Inactive neurons point to the auditory–evoked phasic activation by both the Start and Stop cues (in Start/Stop and Start tests). Times from −1.5 to −0.5 s before the Initiation epoch were always used as the baseline. **(B)** Histogram for each test of the percentage of neurons that sustain an Active or Inactive modulation during licking. **(C)** Histogram showing the percentage of Lick-Active or Lick-Inactive neurons with a significant phasic modulation at the Start cue. **(D)** The proportion of Lick-Inactive neurons that responded phasically to both the Start (Click) and Stop cues (white noise). Horizontal lines in **(C,D)** indicate significant differences among tests at an alpha of 0.05 (chi-squared test).

A total of 421, 304, and 265 NAcSh neurons were recorded during the Gustatory, Start, and Start/Stop brief-access tests, respectively (The representative location of the recording site can be seen in Figure S1 and details about the number of sessions recorded per subject can be found in Table S1). Below we describe and characterize five different NAcSh neuronal populations (some mutually exclusive and some overlapping; Figure S3 depicts the actual numbers and statistics) that were modulated while the animals were licking. We will first explain the Palatability-related responses (Figures 4A–D), and then the Concentration-related neurons (Figures 4E–H), followed by a population of neurons that fired in synchrony with licking (Figure 5). Finally, and according to their modulation during licking, we characterize the two major groups: The Lick-Active and Lick-Inactive (Figure 6). Although these two groups are mutually exclusive, they can be composed of members of the Palatability, Concentration, and Lick-Coherent neurons (Figure S3).

#### A population of NAcSh neurons tracks oromotor sucrose palatability responses and a distinct population tracks sucrose concentration

**Figure 7:**
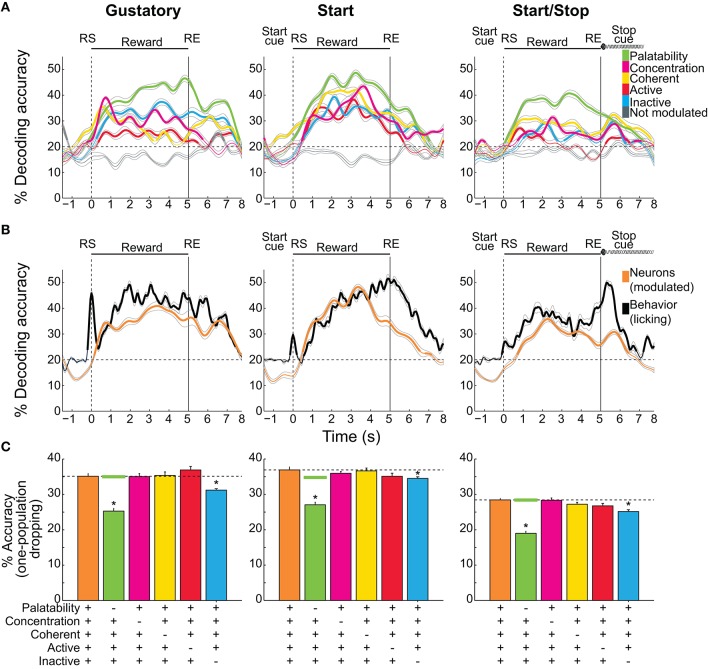
Decoding accuracy of sucrose concentrations/palatability by different NAcSh populations. **(A)** The temporal decoding dynamics in the three brief-access tests of the five sucrose concentrations (palatability) achieved by the five distinct populations. A sixth group is comprised of neurons that do not belong to any population were named “Not modulated” (gray lines). Values are the average and sem of single trials that were correctly classified around RS (time = 0 s). Thicker lines in all panels indicate significantly above chance level (for five stimuli = 20%, horizontal dashed lines) as ascertained by a one group right-tailed t-test (*p* < 0.05). **(B)** The time course of the decoding accuracy of sucrose concentrations/palatability achieved by pooling all groups (all modulated neurons in orange) and the decoding reached by the licking responses per se (black; oromotor palatability). **(C)** Overall decoding during the Reward epoch (mean ± sem) in the three tests as a function of all the Modulated neurons (orange bars and horizontal dashed lines) and the same group but without the participation of each of the populations. Horizontal green lines are the average levels of decoding achieved by dropping (100 times) a random population of neurons as large as the Palatability population. ^*^means significant differences with the Modulated population at the alpha level of 0.05.

To identify whether NAcSh neurons contain information about sucrose's palatability, an analysis was developed that detects the “best time window” where the neuronal responses correlate with the licking responses elicited by the concentrations of sucrose (Figures 4A–D and Materials and Methods). In preparation for further results and discussion, we note that the best time window does not necessarily have to be the onset of palatability (for onsets see Figure 7B). Figures 4A,B show two representative examples of Palatability index related responses: one whose activity increased (Figure 4A) and one that decreased (Figure 4B). The neuronal response is shown in Figure 4A had an anticipatory “ramping” activity starting−1 s before the RS whereupon, at 0.3 s, its firing rate tracked the sucrose's palatability. For this response, the time-window that reached the best correlation after RS with the Palatability index (Pearson's *r* = 0.67) was from 1 to 4 s (see in PSTH the green rectangle in Figure 4A). In contrast, the response shown in Figure 4B had a negative correlation with the Palatability index. It is seen that about 1 s after RS the neuron responded best to water trials (i.e., it fired more in the inter-bout intervals) and its activity decreased with increasing sucrose concentrations such that the overall firing rate during the best time-window (2-3 s) anticorrelated with the oromotor responses elicited by increasing sucrose concentrations (*r* = −0.65). The difference in the number of oromotor palatability responses between the Start and the Start/Stop tests reached significance (Figure 4C; chi-squared test; χ^2^ = 3.93, *p* = 0.047) but did not between other comparisons among brief-access tests (χ^2^ = 2.12, *p* = 0.14, for Gustatory vs. Start; and χ^2^ = 0.56, *p* = 0.45, for Gustatory vs. Start/Stop). Moreover, the population responses of all Palatability index-related neurons recorded in the three tests tracked the trial-by-trial expression of the rat's sucrose palatability (Figure 4D; *r* = 0.30 and *r* = −0.35, *p*'s < 0.01 respectively; no significant differences were observed among brief-access tests; data not shown). These data revealed the presence of an accumbal neural population that tracks the Palatability index elicited by sucrose.

Having identified a population of NAcSh neurons that correlate with sucrose's palatability, we searched for another population that is sucrose concentration-dependent but palatability-independent. For this reason, we analyzed only Complete trials, where the lick rate was similar across sucrose concentrations (Figure 1). Examples of such neuronal responses are seen in Figures 4E,F where the firing rate increased or decreased, respectively with sucrose concentration in a manner that was independent of oromotor palatability. Overall, the activity of these neurons correlated with the increase in sucrose concentrations (*r* = 0.43 and *r* = −0.45; *p*'s < 0.01; Figure 4H). The percentage of concentration-related neurons was not significantly different among behavioral tasks (Figure 4G; chi-squared test; χ^2^ = 0.03, *p* = 0.86, for Gustatory vs. Start; χ^2^ = 0.32, *p* = 0.57, for Gustatory vs. Start/Stop; and χ^2^ = 0.14 *p* = 0.7 for Start vs. Start/Stop), suggesting that the encoding of sucrose's concentrations in the NAcSh is independent of these different behavioral contexts. In addition, neurons only carrying information about sucrose concentrations were less frequently observed than palatability-related neurons (297 Palatability-related neurons vs. 138 concentration-related neurons; chi-squared test; χ^2^ = 47.8, *p* < 0.001). These data demonstrate that the NAcSh contains a licking rate independent neural population that tracks the sucrose-concentrations.

#### A synchronous palatability code for sucrose concentration

We have previously identified NAcSh neurons that fired in phase with the rhythmic tongue protrusion-retraction cycle (Gutierrez et al., 2010). Here we determined if their correlation with licking (coherence; C) contains information about the sucrose concentration and/or somatosensory/motor information. By definition, the coherence ranges between 0 and 1 (with 0 meaning no relation between spikes and lick frequency, and 1 meaning a perfect correlation in both phase and frequency). Table 3 contains, for each brief-access test, the percentages of neurons that were significantly entrained with licking. From these data, it is seen that 8.5% (84/990) of NAcSh neurons were coherent with licking and that their coherence did not change across behavioral tests (total mean for the three tests C = 0.44). We found that the majority of coherent neurons did not discriminate among sucrose Trial Types, and thus contain somatosensory/oromotor information. An example of this kind of response is seen in the upper PSTH in Figure 5A. In contrast, a subpopulation of coherent neurons changed their coherence as a function of the sucrose concentration (Figure 5A lower, Figure 5B, and Table 3). The vast majority of these neurons (*n* = 12) increased their coherence with increasing sucrose concentrations, whereas only 2 decreased their coherence (not shown). In sum, a subpopulation of palatability-coherent neurons was identified that tracks the sucrose concentration.

**Table 3:** Coherent neurons in the NAcSh that convey information about sucrose-concentration.

	**All coherent neurons**		**Coherent and taste-coding**	**Direction of modulation**	
	***n* (%)**	**Coherence mean±sem**	***n* (%)**	**Increased**	**Decreased**
Gustatory (*N* = 421)	32(7.6)	0.46 ± 0.01	5/32(15.6)	4	1
Start (*N* = 304)	27(8.8)	0.44 ± 0.01	4/27(14.8)	3	1
Start/Stop (*N* = 265)	25(9.3)	0.43 ± 0.01	5/25(20)	5	0

*The N's are the total number of neurons recorded in each test. For “All coherent neurons” n's are the number (%) relative to total neurons. For “Coherent and taste-coding” the (%) are relative to all coherent neurons detected. The coherence values are presented as mean ± sem. The number of taste-coding neurons that Increased or Decreased their coherence as a function of sucrose concentration is also shown*.

#### Modulation of lick-active and lick-inactive NAcSh neurons during the reward epoch

As noted, the two most frequent modulations reported in the NAcSh when an animal is licking include one (smaller) group that is activated and a (larger) group that is inactivated (Roitman et al., 2005; Krause et al., 2010; Tellez et al., 2012). Figure 6A shows the population activity of NAcSh neurons that are organized according to their responses preceding and during licking. These are called Lick-Active (red) and Lick-Inactive (blue). Figure 6B shows the percentages of Lick–Active (~18%) and Lick-Inactive (~40%) responses. These two neuronal modulations were equally observed in the three brief-access tests (Figure 6B; *p* > 0.05 n.s.). The Lick-Active and Lick–Inactive responses were aligned to three-time points: Initiation onset (or Start cue; left column Figure 6A), the RS (middle column), and the RE (right column). The upper panel depicts the population activity of neurons recorded in the Gustatory test (control- no auditory stimuli) and the middle and lower rows for the Start and Start/Stop tests, respectively. In the Gustatory test no auditory response was observed, but when responses were aligned to the RS, both the Lick-Active and Lick-Inactive neurons ~ 0.3 s preceding lick onset (RS) their activity either increased or decreased, respectively. Some of the Lick-Active neurons also displayed at least two oscillatory peaks phase-locked to the rat's tongue making contact with the sipper (with the second peak being largest). These oscillations gradually decreased as the “jitter” in the lick rate accumulates (see each lick cycle in the gray lines). When responses were aligned to the RE, it is seen that the population activity of the Lick-Active neurons still maintained their tonic activation throughout the entire Reward epoch and slowly returned to baseline as the animal stopped licking (Empty licks epoch). For the Lick-Inactive neurons, their inhibition began before (RS) and continued to increase until it plateaued at 0.5 s after RS. At the RE, as the animal stopped licking the empty sipper, the activity of the Lick-Inactive neurons did not completely return to baseline activity even after 1.5 s (z-score = 0).

#### Lick-inactive neurons track palatability and play a sentinel role during sucrose consumption

Given that the animals respond behaviorally to auditory cues (Figures 2, 3D), we next explored whether these auditory responses are present in these two major NAcSh groups. Lick-Inactive neurons contain oromotor-palatability information (Figure 7), and most were responsive to auditory stimuli. Specifically, in comparison with the Lick-Active neurons in the Start and Start/Stop tests, the Lick-Inactive neurons more frequently responded with a phasic activation to the auditory Start cue [χ^2^ = 8.08, *p* = 0.004 (Gustatory test); χ^2^ = 42.08, *p* < 0.0001 (Start test) and χ^2^ = 23.5, *p* < 0.0001 (Start/Stop test)]. Figure 6C shows that in the Start test 24.7% (75/304) of neurons were both Lick-Inactive and responsive to the Start auditory cue. That is, 60.4% (75/124) of all Lick-Inactive neurons (in this test) exhibited a significant phasic activation at the Start cue and from this subset 45% (34/75) were also palatability-related neurons.

In the Start/Stop test, only 14.3% (38/265) of the total number of neurons recorded were both Lick-Inactive and also responsive to the Start cue (Figure 6C, and the arrow in Figure 6A). They represent 33.3% (38/114) of Lick-Inactive neurons and 50% (19/38) correlate with sucrose palatability. Furthermore, 60.5% (23/38; Figure 6D) of the above-mentioned Lick-Inactive neurons also responded with a phasic activation to the Stop cue (see arrow Figure 6A right panel), and of this 56.5% (13/23) tracked the sucrose palatability. These findings revealed that a subpopulation of the Lick-Inactive neurons could respond to both auditory and palatability related information to track feeding behavior.

In contrast to Lick-Inactive neurons, Lick-Active neurons are unresponsive to auditory cues but abruptly return to baseline activity after the reward ends (Figure 6C). That is only ~ 1–4% (6/421 in the Gustatory, 11/304 in the Start, and 5/265 in the Start/Stop test) of Lick-Active neurons responded to the auditory Start cue (Figure 6C). However, in the Start/Stop test, at the Stop cue the Lick-Active neurons abruptly decreased their firing rate, and then rebounded before finally returning to baseline levels (Figure 6A). Likewise, at the Stop cue, the population activity of Lick-Inactive neurons more rapidly returned to baseline (0.9 s) than in the other two tests (>1.5 s for Gustatory and Start test). These rapid modulations in the Start/Stop test observed in both Lick-Active and Lick-Inactive neurons could be a factor as to why in the Start/Stop test rats stopped licking the empty sipper faster.

#### Decoding of sucrose concentrations/palatability by different NAcSh populations

Having described different NAcSh populations that carry information about sucrose's palatability, its concentrations, and licking, we investigated, during the Reward epoch, the amount of information about sucrose (measured as the percent accuracy of trials correctly classified) that each population contains and their temporal decoding dynamics. Figure 7A shows the time course of decoding accuracy in each behavioral test as a function of the following NAcSh populations: Palatability-related (Figures 4A–D), Concentration-related (Figures 4E–H), Coherent (Figure 5), Lick-Active (Figure 6), and Lick-Inactive (Figure 6). The remaining neurons that do not belong to any of the latter groups were named, “Not modulated.” We are aware that this analysis does not distinguish palatability information from sucrose concentration and thus reflects the overall information available about sucrose (palatability/sucrose concentration combined). As a control, the “Not modulated” neurons (gray lines) in the three tests did not decode better than chance, suggesting that the information about sucrose available in the NAcSh was in the Modulated neurons (right-tailed *t*-test, *p*'s < 0.05; see ticker lines in Figure 7A). In this regard, in the three tests, the Palatability-related neurons (green in Figure 7A) always had more information about sucrose palatability/concentrations, whereas the Lick-Active neurons yielded the least information.

Our analysis revealed that the decoding performance was test–dependent. That is, the decoding was better and was more similar among NAcSh populations in the Start test, while in the Start/Stop test the decoding was less and more heterogeneous (Two-way ANOVA; main effect of neural population *F*_(2, 4)_ = 215.9, *p* < 0.0001; main effect of tasks, *F*_(2, 4)_ = 262.5, *p* < 0.0001). Specifically, the decoding was better in the Start task followed by the Gustatory test, and these two were better than the Start/Stop test (all *p*'s < 0.0001). Moreover, there were significant differences among all NAcSh populations (*p*'s < 0.01; except between Coherent vs. Concentration (*p* = 0.067) and Lick-Inactive vs. Concentration populations (*p* = 0.25). Despite the fact that Coherent neurons are a small group, they decoded sucrose information to similar levels as other larger NAcSh populations (yellow; Figure 7A), suggesting that the synchrony code implemented by this population encode gustatory/palatability information even in different behavioral contexts.

The overall neuronal and behavioral decoding of sucrose concentration/palatability is shown in Figure 7B. In all three tests, behavioral decoding was slightly superior to neural decoding. However, it is seen that decoding of all Modulated neurons (orange line) preceded (in the Start test) and it follows (in the Start/Stop test) the decoding achieved by just using the licking responses (black lines). We also tested if there was a correlation between the decoding of all Modulated neurons, against the decoding achieved by the lick rate *per se* (Figure 7B). We found that in the three tests the neural decoding significantly correlated with the decoding obtained by the licking responses (Pearson's *r* = 0.87, *r* = 0.8, and *r* = 0.86, for the Gustatory, Start, and Start/Stop tasks, respectively, all *p*'s < 0.001). The “Not modulated” responses did not correlate with licking (*r* = −0.25, *r* = −0.06, and *r* = 0.03 respectively, all *p*'s > 0.05). These data suggest that only feeding-modulated NAcSh neurons track sucrose nearly as well as licking palatability responses.

We then evaluated the temporal aspects of the onset of sucrose palatability in the animals' behavior and on the NAcSh activity. Figure 7B shows the onsets where all Modulated NAcSh neurons (orange lines) could correctly classify Trial Types. These onsets differed among tests being 0.15 s for the Gustatory, 0.5 s in the Start, and 0.7 s in the Start/Stop test. For the licking responses in the three tasks (black lines), the onset of behavioral decoding began almost immediately after the RS (< 0.15-0.3 s, in 1 or 2 licks), suggesting that the expression of palatability is rapid. We note that the significant decoding above the chance level before RS was due to the sliding window procedure (see Materials and Methods). To prove this point, we repeated the same analysis, but this time with no sliding window and, as expected, no significant decoding was found before time = 0 s (data not shown). Interestingly, in the Start test, the lick rate rapidly decoded information about sucrose's concentrations. That is, decoding accuracy quickly started in less than two licks, but then it dropped to chance level ~0.5 s after the RS. A similar, but less marked fall in decoding was also seen in the Gustatory task, although the pause in licking observed in both the Gustatory and the Start test could rationalize this effect (see Figure 1B inset, Figure 2A). In summary, we showed that palatability is a phenomenon that rapidly appears and gradually grows across the Reward epoch. We also found that auditory cues affect not only the behavioral expression of sucrose palatability, but also its NAcSh neural decoding.

We next explored which of the neuronal population(s) contributed the most to the neural decoding of the five sucrose concentrations. This was achieved by performing a one-population dropping analysis (Figure 7C). For this analysis, during the Reward epoch the decoding performance was calculated both for all Modulated neurons and when each population was removed (dropped). In comparison with the decoding of Modulated neurons in the three tests (Gustatory 35%, Start 36.9%, and Start/Stop 28.4% accurate), we found that when the Palatability population was excluded from analysis the neural decoding accuracy significantly fell near to chance level (20.3, 27, and 19% accurate; Kruskal-Wallis test; all *p*'s < 0.05). It also decreased when the Lick-Inactive population was dropped (31.2, 34.5, and 25.1%; all *p*'s < 0.05). Dropping other populations did not impair performance. We note that removing the Palatability neurons always lead to a larger impact (decrease) in the overall sucrose decoding. Given that the number of Palatability-related neurons was always smaller than the Lick-Inactive neurons, then it is unlikely that the impairment in neural decoding was due to a simple reduction in the population size. Nevertheless, to further prove that the dropping in performance was not due to chance, we repeated the same analysis but now removing randomly (from Modulated neurons) the same number of neurons as in the Palatability population. This analysis was repeated 100 times, and the averaged random performance (Figure 7C -green horizontal line) was compared against the decoding of all Modulated neurons (the orange bar and horizontal dashed lines in Figure 7C) and against to the Palatability-related population dropped from the analysis (see a green bar in Figure 7C). No differences in decoding was found between all Modulated neurons and the dropping of the randomly-selected neurons (which did not affect the neural decoding 35, 34.8, and 28.4% for each test; compare horizontal dashed lines with green lines in Figure 7C; all *p*'s > 0.05). In contrast, there were significant differences in decoding between dropping a random population and removing the Palatability-related neurons (all *p*'s < 0.05), thus the information contained in the Palatability and the Lick-Inactive population was not due to chance. In summary, Palatability-related and Lick-Inactive neurons, provide unique information about predicting sucrose palatability/concentration that cannot be compensated by any other NAcSh population.

#### Dynamic tracking of palatability over the entire course of a behavioral session

**Figure 8:**
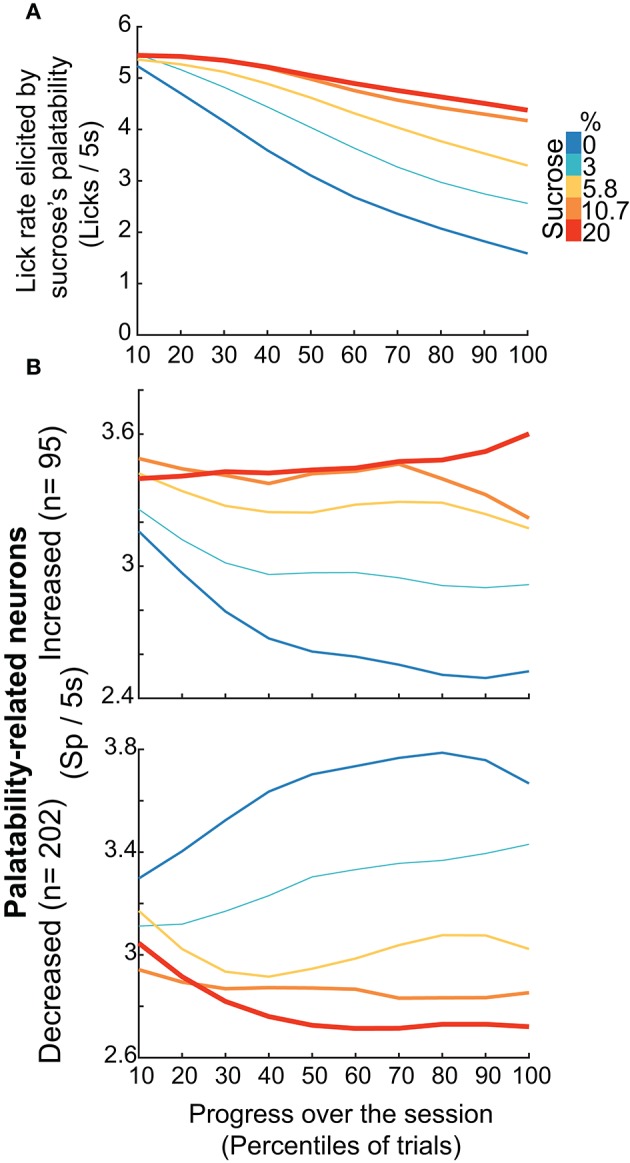
NAcSh Palatability-related neurons dynamically track the changes in lick rate elicited by sucrose's palatability over the course of the session. **(A)** It depicts the population PSTHs of the lick rates in the Reward epoch (Licks/5 s) as a function of Trial Types, divided into blocks of 10th percentile of trials each. The lick rate declined over the course of the session in a concentration dependent manner [Repeated Measures-ANOVA, main effect of Trial Types, *F*_(4, 915)_ = 86.5, *p* < 0.0001, effect of time, *F*_(4, 9)_ = 474.06, *p* < 0.0001, and significant interaction Trial Types × time, *F*_(36, 8235)_ = 20.9, *p* < 0.0001]. **(B)** Population PSTHs of the firing rate during the Reward epoch (spikes/5 s) of the Palatability-related neurons over the course of the session for each Trial Type. The upper panel depicts neurons that fired more to higher concentrations, whereas lower panel shows neurons with decreasing firing rates as the sucrose concentration increased. Similar results were found by using only the firing rate during the “best-window” of each neuron (see Figure S4).

Having demonstrated that, regardless of the brief-access test, the Palatability-related neurons exhibited the most information about sucrose's concentration, we then explored whether this population can dynamically adjust their responses to track the changes in sucrose's palatability over the course of the session when the animals would approach satiation. This was accomplished by combining, during the 5 s Reward epoch in the three tests, all the Palatability-related neurons recorded, and then computing the lick rates and the population-firing rate. Then we plotted the activity across the session every 10th—percentiles of trials, guaranteeing that every block across the session has the same number of trials. We found that within the session the lick rate declined in a concentration-dependent manner. That is, early in the session rats licked more similarly for all concentrations, but as the session progressed, the lick rate declined more and more rapidly for lower concentrations, especially for 0 and 3% thus indicating palatability rather than satiety. Likewise, the neural activity of all Palatability-related neurons (either increasing -upper- or decreasing -lower panel) tracked over the course of the session and in a sucrose-concentration dependent manner the oromotor responses elicited by sucrose ingestion (Figure 8B). In fact, the lick rate correlated with neural responses in the 0% (Increased, *r* = 0.9; Decreased, *r* = −0.9; *p*'s < 0.0001) and 3% trials (Increased, *r* = 0.67, *p* = 0.034; Decreased, *r* = −0.88; *p* < 0.0001) but not for other Trial Types (*p*'s > 0.05). Thus, as the animals approach satiety the responses of the Palatability-related neurons tracked the rapid decline in lick rates observed over the course of the session for the two lowest concentrations (0 and 3%), suggesting they are tracking palatability and not satiety.

#### Phasic inhibitions after the reward end

**Figure 9:**
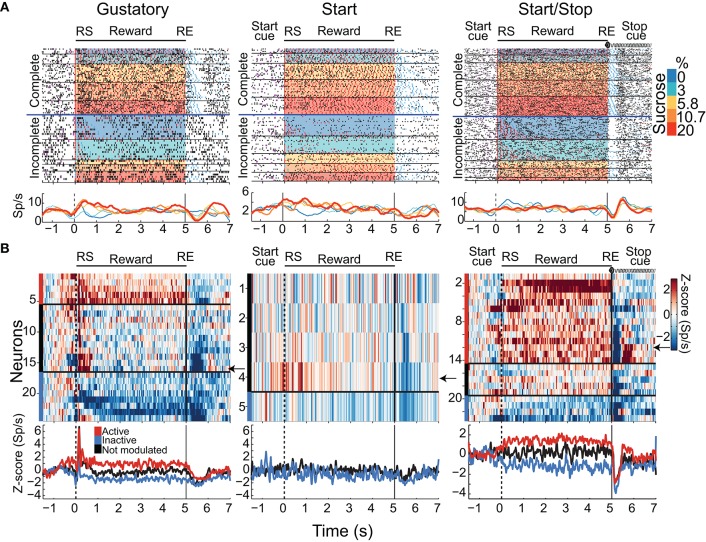
A population of NAcSh neurons signals the Reward End (RE) by transiently inhibiting their activity after the first available cue signal that the Reward epoch has ended. **(A)** Raster plots of representative neuronal responses aligned to the RS of neurons recorded in the Gustatory (left), the Start (middle), and Start/Stop (right) tests that, after the reward end (RE), display a transient inhibition. Note that in both Complete and Incomplete trials (separated by a horizontal blue line) the inhibitions in the Start/Stop task were more phase-locked to the RE. **(B)** The color-coded normalized Z-scores, using a baseline −1.5 to 0.5 s relative to and aligned with RS of all neurons with a phasic inhibition after RE and their corresponding population PSTH below (*n* = 24, 5, and 23 for Gustatory, Start, and Start/Stop test, respectively). Neurons were characterized as a function of their modulation profile relative to licking: Lick-Active, Lick-Inactive, and Not modulated. Black horizontal lines in the upper panel separate the three groups. On the left side, the vertical bars in color indicate their membership to each subgroup with the same conventions as in Figure 6. The arrow indicates the example shown in rater plot in **(A)**.

In addition to phasic activations evoked by the Stop cue described above, we also recorded neurons that phasically paused their firing activity after the RE. For this reason, we explored whether some of these phasic inhibitory responses would track the absence of a reward (Apicella et al., 2009). Examples of such types of neuronal responses are seen in Figure 9A. The left panel shows a raster plot and corresponding PSTH of a neuronal response recorded in the Gustatory task, that after RE, decreased its firing rate starting at RE + 0.3 s and maintained this inhibition for an additional 1 s. Note that the inhibition began after RE and after two or three empty licks, and always preceded the stopping of licking, suggesting that it may track the absence of reward (after RE). The middle panel shows a neuronal response from a Start test, which becomes inhibited within the first two empty licks but with a much smaller magnitude than seen in the Gustatory test. The right-hand panel shows a raster plot of a response from a Start/Stop test. These responses exhibited the sharpest inhibitions immediately after the Stop cue. After the phasic inhibition, they mostly exhibited a rebound in activity (also see Figure 9B right color panel for all neurons). The onset of inhibition always preceded any empty licks (in both the Complete and Incomplete trials), suggesting that, in this case, the auditory Stop cue (and not the absence of reward) evoked the inhibition. It follows that for rats trained in the Start/Stop test, the Stop cue was more informative about stopping licking than the absence of liquid reward.

Figure 9B shows, for the three tests, the color-coded (upper) and PSTHs (lower) of all the neurons exhibiting a significant phasic inhibition. Their activity was normalized to the baseline period, aligned to the RS (time = 0 s), and was sorted as a function of their licking-modulation profile (the red vertical bar indicates the Lick-Active neurons, black for Not modulated (by licking), and blue for Lick-Inactive). Since all are phasically inhibited after or at the RE, this modulation was treated as a single group. The percentage of neurons was different among tasks: 5.7% (24/421), 1.6% (5/304), and 8.6% (23/265) in the Gustatory, Start, and Start/Stop tasks, respectively. They were less commonly found in the Start test than in the other two tests (All Chi-squared *p*'s < 0.01). As noted, they also differed in their onset of inhibition: the mean (± sem) was 0.29 (± 0.05), 0.44 (± 0.16) and 0.16 s (± 0.03) for the Gustatory, Start, and Start/Stop test, respectively [one-way ANOVA; *F*_(2, 47)_ = 6, *p* < 0.01], being the Start/Stop modulations the fastest ones in comparison with the other two tests (all *p*'s ≤ 0.01). Thus, these phasic inhibitions (along with the phasic activations at the Stop cue see Figure 9A Start/Stop and right panel) can rationalize why animals cued with a Start/Stop signal were more rapid to stop licking an empty sipper.

## Discussion

Sucrose's palatability is a primary reason for its excessive intake and one culprit of the worldwide obesity epidemic (Popkin and Nielsen, 2003). Although satiety/hunger signals control feeding, food intake can be influenced by external food cues (Weingarten, 1984; Nitschke et al., 2006; Spence, 2015; Livneh et al., 2017). We investigated in behaving animals how external auditory stimuli enhances sucrose palatability together with its responses in the NAcSh, a limbic area involved in reward, feeding, and sensory/motor transformations (Roesch et al., 2009; McGinty et al., 2013; West and Carelli, 2016). The goals of this study were to determine whether NAcSh responses would track sucrose's palatability (licking responses), its concentration and, in addition, how they process auditory information. In behavioral studies, we found that Start and Stop auditory cues can differentially enhance sucrose palatability to the extent that when both cues are given, the animals feed continuously and increase their caloric intake. In agreement with previous studies, we found that when animals were licking two primary NAcSh responses, called Lick-Active and Lick-Inactive, were evoked (Roitman et al., 2005; Krause et al., 2010; Tellez et al., 2012). We also identified several distinct populations of NAcSh responses that tracked either the licking responses elicited by sucrose's palatability or the sucrose concentrations by increasing or decreasing their activity. Another, not previously described NAcSh population, fired in synchrony with licking and exhibited an enhancement in their coherence with increasing sucrose concentrations. Another finding was that only the Lick-Inactive neurons were phasically activated by both auditory cues, suggesting that they play a sentinel role monitoring relevant food-related cues to increase hedonically positive oromotor-responses elicited by sucrose's palatability and its caloric intake.

Several studies have shown that exteroceptive stimuli can modulate feeding (Weingarten, 1984; Spence, 2015). In particular, the auditory atmosphere in a restaurant can influence flavor perception, and perceived ethnicity of a dish (Spence, 2015). Here we have added to this literature by showing that sucrose's palatability responses can be enhanced either by an auditory cue that anticipates the delivery of a reward or by two auditory cues that announce both the start and end of the Reward epoch (Figures 2, 3). Moreover, we identified the neural correlates of these responses in the NAcSh. For the behavioral responses, the auditory Start cue enhanced palatability by altering the expectancy of the onset of a reward and the Start/Stop cue by allowing animals to freely lick until the Reward epoch ended. Since the Start-cue enhanced sucrose palatability (Figure 7 and Table 2), this result is consistent with a study showing that an anticipatory auditory cue associated with the intra-oral delivery of sucrose can enhance the palatability of water (Delamater et al., 1986). We extended these observations by showing that sucrose's palatability (Figure 2 and Table 1 Tastant/Water ratio) can be enhanced by a general expectancy Start cue. Also shown was that the onset of palatability could be slowed by a signal that allows animals to lick until a Stop cue announces the termination of a reward (Figure 7B). Finally, we demonstrated that the Start/Stop auditory cues could increase caloric intake (Figures 3B,C), indicating that exteroceptive food cues (either auditory or visual) may play a contributory role in an obesogenic environment (Schachter, 1971; Sample et al., 2015; Spence, 2015; Livneh et al., 2017).

### Palatability- and sensory-related information in the NAcSh

Although many studies have related NAcSh responses to the palatability of hedonically positive and negative tastants (Roitman et al., 2005; Carlezon and Thomas, 2009), remains no consensus about how the NAcSh tracks the hedonic and/or sensory properties of sucrose (Roitman et al., 2005; Taha and Fields, 2005). As noted, Roitman et al. (2005) suggested that NAcSh inhibitions were tuned to the reward of sucrose, whereas their activations were related to the aversion elicited by quinine. Thus, this type of neuronal modulation then correlates with the different palatability oromotor-responses elicited by appetitive and bitter tastants. A different scenario was proposed by Taha and Fields (2005), using only a sucrose task. They found that the majority of NAcSh neurons, which are inactivated during consumption of sucrose (here referred Lick-Inactive) encode appetitive behaviors, whereas the Lick-Active neurons primarily encode the palatability/reward aspects of sucrose. In contrast, here we identified a population of NAcSh neurons that tracks sucrose palatability with either a positive or a negative correlation (Figures 4A–D). In fact, many of the Palatability-related neurons also belong to the Lick-Inactive category (Figure S3) and the majority anti-correlated with sucrose palatability (Figure 4B). In addition, Lick-Inactive neurons decoded sucrose information better than the Lick-Active ones (Figure 7A, blue vs. red). Furthermore, the population dropping analysis revealed that the Palatability-related neurons, followed by the Lick-Inactive, were crucial for the neural decoding of sucrose palatability (Figure 7C). These results indicate that these two populations contain unique information about predicting sucrose palatability In addition, we showed for the first time that Palatability-related neurons could dynamically track sucrose's palatability over the course of the session (Figure 8). In sum, we showed that NAcSh palatability-related neurons include a larger and more heterogeneous group than was previously assigned (Roitman et al., 2005; Taha and Fields, 2005).

Our results are consistent with those of Taha and Fields (2005) who found that the magnitude of the inhibition of the Lick-Inactive neurons *per se* does not discriminate sucrose concentrations (see raster plot Figure 4B; from 0 to 1 s). However, because in the Reward epoch, animals stopped licking faster for lower concentrations (less palatable) and continued licking for high sucrose (more palatable), then the firing rate of Palatability-decreasing and Lick-Inactive neurons anti-correlated with the sucrose's palatability responses by increasing their activity as the animals stopped licking. In fact, it is the property of less palatable tastants (including water) to stopping licking that gives better correlations with the NAcSh activity. Our results can be reconciled with those of Taha and Fields (2005) and Roitman et al. (2005) in the sense that they can explain why quinine [an aversive stimulus that rapidly stops rats from licking (Weiss and Di Lorenzo, 2012; Perez et al., 2013)] activated NAcSh neurons, whereas sucrose inhibited them (Roitman et al., 2005). That said, we acknowledge that in this study we have not tested quinine since it would introduce the confounding factor of fear (Perez et al., 2013). In sum, the NAcSh Palatability-related neurons track licking motor-related attributes of palatability (Mogenson et al., 1980; Prado et al., 2016). That said, at this point we can only speculate that neurons in the NAcSh may encode a general palatability signal that can be generalized to other gustatory stimuli different from water and sucrose. This is because, in rodents, palatability is operationally defined as the enhancement of hedonically positive oromotor responses (Berridge and Grill, 1983; Spector et al., 1998) and that this definition should not be confused with a conscious hedonic feeling of pleasant taste that humans feel (Grill and Berridge, 1985; Sclafani, 1991). Moreover, we note that palatability may also depend on the reinforcing value of sucrose, the animals' internal state (Berridge et al., 1984) as well as learned post-ingestional effects associated to tastants (Aravich and Sclafani, 1980; de Araujo et al., 2008). Our experimental design does not allow us to separate the specific contribution of each of these components from the oromotor licking responses elicited by sucrose. For these reasons here we characterize a population of NAcSh neurons that tracks the oromotor- palatability component elicited by sucrose.

Although the NAcSh is not classically included as part of the gustatory pathway (Jones et al., 2006; Simon et al., 2006; Vincis and Fontanini, 2016), our results suggest that this assumption may need revision. The reason is that we identified, for the first time, a distinct group of palatability-independent NAcSh neurons that encode sensory-related information about sucrose's concentrations (Figures 4E–H). These data show that NAcSh neurons not only track palatability information but also receive and relay gustatory information relevant to feeding. The gustatory information may come from sucrose-responsive neurons in the rat parabrachial nucleus (PBN) that preferentially send inputs to the NAcSh (Li et al., 2012). In addition, electrical stimulation of the NAcSh suppressed taste-evoked responses of the PBN (Li et al., 2012), suggesting a PBN-NAcSh loop that can reciprocally modulate the taste of sucrose. Gustatory information can also reach the NAcSh via projections from the medial prefrontal cortex (Hurley et al., 1991), an area involved in taste coding (Jezzini et al., 2013). Here we identified a synchrony code used by a subpopulation of NAcSh neurons (Figure 5) that discriminates the sucrose concentrations. A similar synchrony code has been reported in the invertebrate olfactory system (Bazhenov et al., 2001). Thus, NAcSh neurons not only process palatability information, but a population contains information about sucrose's concentration or intensity.

Another finding of this study regards the temporal dynamics of sucrose palatability. Specifically, in the three brief-access tests used in this study, the onset of the behavioral expression of sucrose palatability was more rapid (<0.15–0.3 s, in 1 or 2 licks; Figure 7B) than the neuronal tracking of palatability. However, after this initial early peak, only in the Start test the NAcSh decoding was better, and it preceded behavioral expression of palatability, indicating that a reward-anticipatory cue enhanced (and accelerated) the encoding of sucrose. This result is in general agreement with previous findings showing that anticipatory exteroceptive cues can decrease the latency of taste coding in the rat gustatory cortex (Yoshida and Katz, 2011; Samuelsen et al., 2012). Furthermore, our auditory Start and Stop cues may have also enhanced positive hedonic responses elicited by sucrose by indirectly changing the predictability of sucrose reward delivery. This, in turn, could change the state of the network into an anticipatory reward state, perhaps recruiting the mesolimbic reward system, as is known that reward-predictive cues develop conditioned DA release in the NAc as learning progress and enhance excitatory synaptic strength in midbrain dopaminergic neurons (Stuber et al., 2008; Edwards et al., 2017; Yang et al., 2018). However, this remains to be experimentally tested.

### Phasic modulations (activations and/or inhibitions) after the reward end correlate with sucrose feeding termination

Given that we found behavioral evidence that rats can use a Stop auditory cue to terminate feeding rapidly, we explored its neuronal correlates in the NAcSh. In this regard, we found a group of neurons with phasic activations (Figure 6) and inhibitions (Figure 9). We posit that both modulations provide information to rats for them to stop licking faster (Figure 3D). Moreover, we propose that the deeper and sharpest the phasic inhibition, such as those observed in the Start/Stop test, the faster the animals will stop feeding, perhaps via disinhibition of MSNs (Figure 6A Start/Stop right panel; English et al., 2012). Such rapid neuronal responses observed in the NAcSh evoked by auditory stimuli are possible (Roitman et al., 2005), since in rats it takes only ~10 ms for an auditory stimulus to activate the auditory rat thalamus (Shiramatsu et al., 2016) and amygdala (LeDoux, 1992) so that it can modulate the NAcSh (Millan et al., 2017).

### Lick-inactive NAcSh neurons play a sentinel (gate) role during sucrose feeding

It has recently been found that the NAcSh is phasically activated by arousing auditory stimuli (Solis et al., 2017). Here we found that the Lick-Inactive neurons (and not the Lick-Active ones) were phasically activated by both the Start and Stop feeding associated cues (Figures 6C,D). Collectively, these data support the hypothesis of a “sentinel-like” control of feeding in the NAcSh (Kelley et al., 2005; O'Connor et al., 2015), which posits that the activity of MSND1+ neurons allows feeding as long as the auditory stimuli in the environment does not represent a threat (O'Connor et al., 2015). Thus, during feeding, accumbal inhibition of MSND1+ authorize the ongoing behavior, but abrupt increases in its activity quickly abort consumption (O'Connor et al., 2015). Furthermore, the same study also demonstrated that MSND1+ neurons are both Lick-Inactive neurons and projection neurons that mono-synaptically inhibit GABAergic neurons in the lateral hypothalamus (LH). Since LH GABAergic neurons are involved in feeding (Jennings et al., 2015), we propose that Lick-Inactive (presumably MSND1+ according to O'Connor et al. (2015) are neurons that can integrate and broadcast not only palatability but also relevant environmental auditory information to LH GABAergic neurons to control feeding and to modulate palatability responses (Li et al., 2013; Nieh et al., 2015).

## Conclusions

We demonstrated that auditory cues associated with feeding could increase sucrose palatability and caloric intake in a task dependent-manner. In ensemble recordings from the NAcSh, a brain region involved in reward, we identified several neuronal populations that track the behavior and encode (and decode) sucrose's palatability and its concentration. More importantly, Palatability-related neurons, with either increasing or decreasing responses, can dynamically track the changes in the lick rate elicited by sucrose's palatability over the course of the session. Finally, we found that the NAcSh Lick-Inactive neurons contain appetitive, palatability, and auditory cue information that may be relevant to modulate the expression of the oromotor component of sucrose palatability.

## Author contributions

MV, SS, and RG conceived and designed the study; MV and MM collected the data; MM built the microelectrodes; MV and RG analyzed and interpreted the data; MV, SS, and RG wrote the manuscript. All the authors agree with the final version of the manuscript.

### Conflict of interest statement

The authors declare that the research was conducted in the absence of any commercial or financial relationships that could be construed as a potential conflict of interest.

## Supplementary material

Figure S1 Representative locations of recording sites. Schematics (left) of coronal sections of the rat brain highlighting, in red, the areas recorded from all rats. The photograph on the right side shows a representative example of a coronal brain section (~1.2 mm from bregma) where two electrode tracks (upper) and a recording site (see magnification below, arrow) targeting the NAcSh are seen. CC, corpus callosum; aca, anterior commissure.

Figure S2 Extracellular recordings of a Palatability-related neuron that was putatively recorded across three consecutive days. **(A)** The blue waveforms are all the action potentials of a single neuron recorded in the NAcSh during a Start test session. Gray traces are the unsorted waveforms (noise signal) recorded from the same channel. **(B)** The same action potentials (blue points) of the neuronal activity depicted in A within the 55-min session. Values are plotted in a three-dimensional space built with the first two principal components (PCs) of the waveforms vs. time. **(C)** Inter-Spike Intervals (ISIs) histograms (blue bars) of single-unit activity recorded from the same electrode during three consecutive days. Insets depict the average waveform. **(D)** Raster plots (upper panels) and PSTHs (lower) of the neuron shown in C with their responses aligned (Time = 0 s) to the RS. Note that in the three days this neuron was inhibited preceding licking initiation and fired more in the water and 3% sucrose trials. Same conventions as in Figure 4. Briefly, black ticks depict the action potentials as a function of the color-coded sucrose concentrations. The horizontal blue lines separate Complete from Incomplete trials. The licking responses are shown in red ticks.

Figure S3 In the three tests, Palatability-related and Lick-Inactive neurons were more overlapped. Contingency tables showing for each brief-access test the number (%) of neurons that belong to two different NAcSh populations. For each square in the contingency table, a Fisher's exact test was performed. Values in bold indicate that the probability of overlapping was statistically different than chance (Fisher's exact test; *p* < 0.05). The color code means the amount of overlap where 100% corresponds to the total number of neurons in each pair of neuronal populations. The “n” in parenthesis shows the total number of neurons belonging to each population.

Figure S4 The firing rate of the Palatability-related neurons during their “best-window” is sufficient to track the changes in lick rate over the course of the session. Population PSTHs of the firing rate during the “best-window” of each Palatability-related neuron as a function of the progress over the session and sucrose concentration. Upper panel shows neurons with increasing and lower panels with decreasing firing rate as a function of sucrose concentration. Similar results are found using the firing rate in the entire Reward epoch (see Figure 8A).

Table S1 Number of single-units recorded per session.
