# Supplementary figures and images for: Encoding of Sucrose's Palatability in the Nucleus Accumbens Shell and Its Modulation by Exteroceptive Auditory Cues
Source: Front Neurosci. 2018 May 4;12:265. doi: 10.3389/fnins.2018.00265 (PMC5945833; doi:10.3389/fnins.2018.00265)

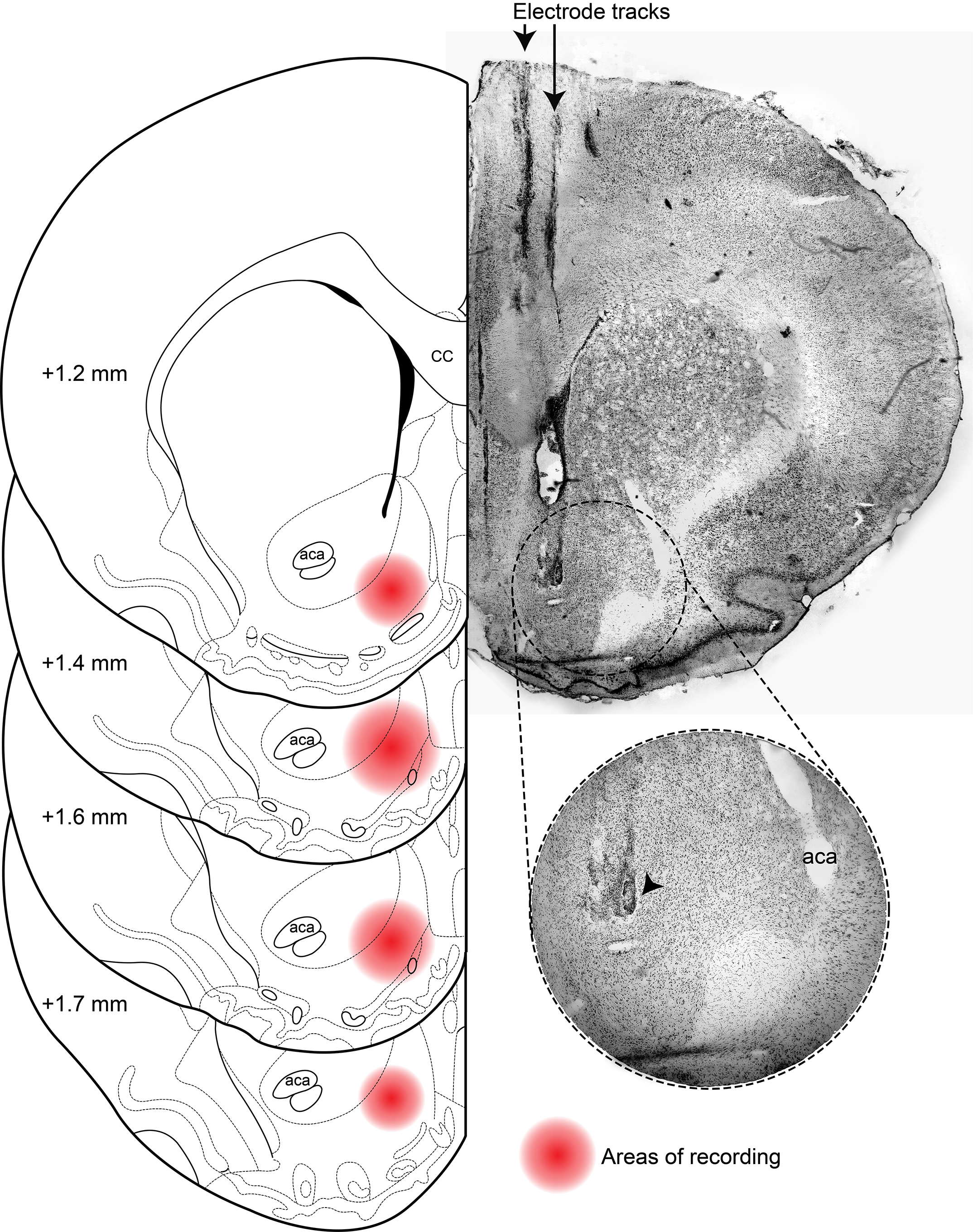

Supplement: Figure S1 — Representative locations of recording sites. Schematics (left) of coronal sections of the rat brain highlighting, in red, the areas recorded from all rats. The photograph on the right side shows a representative example of a coronal brain section (~1.2 mm from bregma) where two electrode tracks (upper) and a recording site (see magnification below, arrow) targeting the NAcSh are seen. CC, corpus callosum; aca, anterior commissure. [file Image_1.TIF]

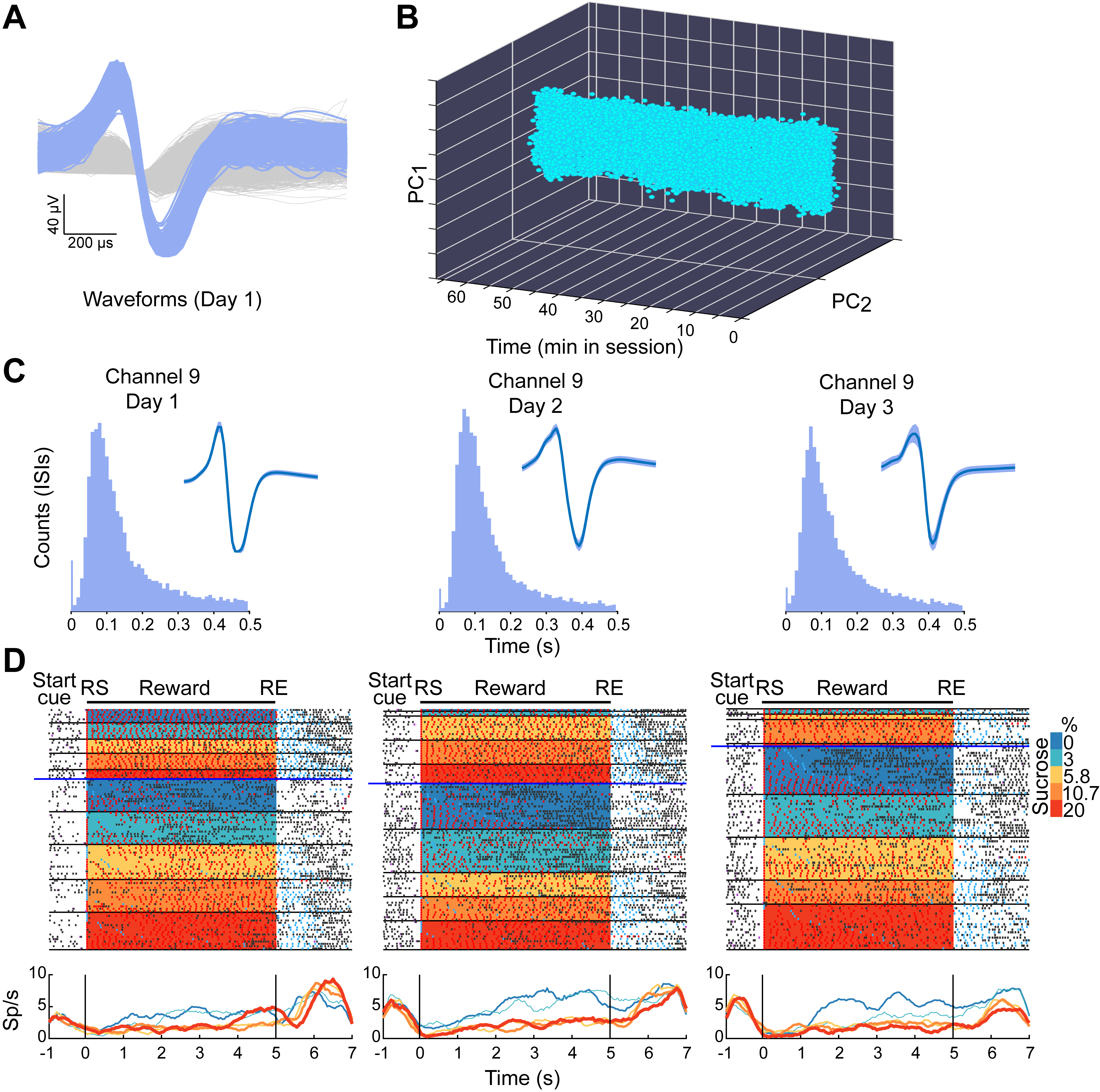

Supplement: Figure S2 — Extracellular recordings of a Palatability-related neuron that was putatively recorded across three consecutive days. (A) The blue waveforms are all the action potentials of a single neuron recorded in the NAcSh during a Start test session. Gray traces are the unsorted waveforms (noise signal) recorded from the same channel. (B) The same action potentials (blue points) of the neuronal activity depicted in A within the 55-min session. Values are plotted in a three-dimensional space built with the first two principal components (PCs) of the waveforms vs. time. (C) Inter-Spike Intervals (ISIs) histograms (blue bars) of single-unit activity recorded from the same electrode during three consecutive days. Insets depict the average waveform. (D) Raster plots (upper panels) and PSTHs (lower) of the neuron shown in C with their responses aligned (Time = 0 s) to the RS. Note that in the three days this neuron was inhibited preceding licking initiation and fired more in the water and 3% sucrose trials. Same conventions as in Figure 4. Briefly, black ticks depict the action potentials as a function of the color-coded sucrose concentrations. The horizontal blue lines separate Complete from Incomplete trials. The licking responses are shown in red ticks. [file Image_2.TIF]

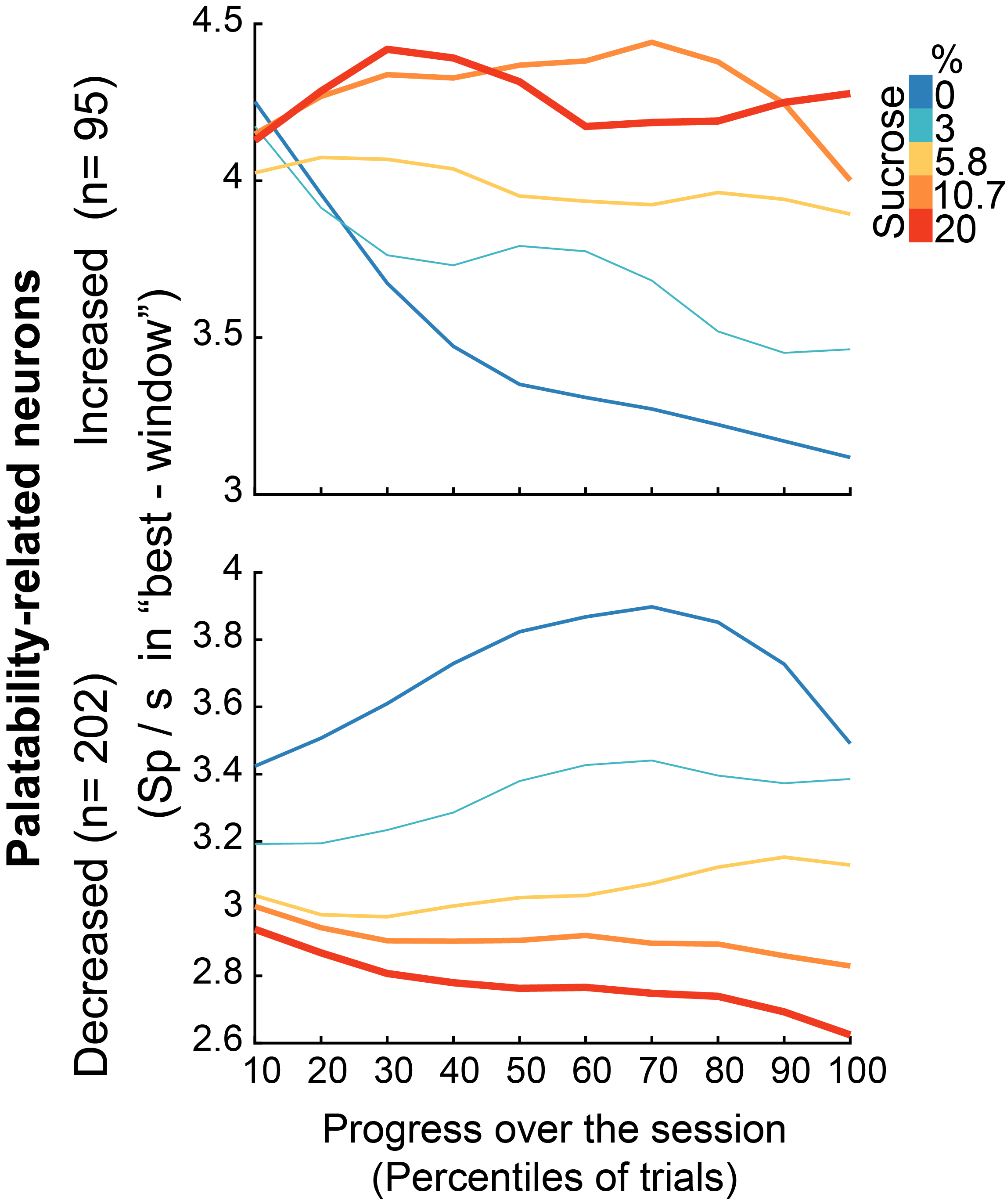

Supplement: Figure S4 — The firing rate of the Palatability-related neurons during their “best-window” is sufficient to track the changes in lick rate over the course of the session. Population PSTHs of the firing rate during the “best-window” of each Palatability-related neuron as a function of the progress over the session and sucrose concentration. Upper panel shows neurons with increasing and lower panels with decreasing firing rate as a function of sucrose concentration. Similar results are found using the firing rate in the entire Reward epoch (see Figure 8A). [file Image_4.TIF]
