# Supplementary material for: Encoding of Sucrose's Palatability in the Nucleus Accumbens Shell and Its Modulation by Exteroceptive Auditory Cues
Source: Front Neurosci. 2018 May 4;12:265. doi: 10.3389/fnins.2018.00265 (PMC5945833; doi:10.3389/fnins.2018.00265)
Supplement: Table S1 — Number of single-units recorded per session. [file Data_Sheet_1.docx]

**Overlapping of neuronal populations**

To quantify the amount of overlap between the NAcSh neuronal populations modulated in our tasks (Palatability, Concentration, Coherent, Active and Inactive) we constructed for each brief access test a contingency table (Fig. S3). They contained, in each location, the number (%) of neurons that belong to both NAcSh populations in that intersection. 100 % is the total number of neurons (without repetition) belonging to both populations. Then, we determined, for each location in the contingency tables, if the probability of a neuron to belong to both populations statistically was due to the chance by using the Fisher's exact test (two tails, α = 0.05) (Agresti, 2007).

**Statistical tests**

All data processing and statistical analysis were performed using MATLAB (The MathWorks Inc., Natick, MA) and StatView 4.57 (Abacus Concepts, Inc., Berkeley, CA). To calculate the Pearson's correlation coefficient r between the lick rate (in the Reward epoch) and Trial Types, we pooled the lick rates in each trial across sessions and subjects. Distributions of the lick rate and first lick bout duration were tested with a two-way ANOVA by combining trials across sessions and subjects. The behavioral tests were set as Level 1 (with three factors = the three brief-access tests) and Trial Types as Level 2 (with five factors = sucrose concentrations). A Fisher’s LSD *post hoc* was then performed. The correlations of the first lick bout duration and Trial Types were calculated from all trials of each behavioral test and subjects. Comparisons of the proportions of single lick bouts among tasks were analyzed using chi-squared tests (contingency table). To compare differences in lick rate, first bout duration, Bout index, and kcal across tasks and sucrose concentrations, we used a two-way ANOVA. We used the behavioral tests and Trial Types as Levels with three and five factors, respectively, except for calorie intake that contains only four concentrations in the Trial Type factor with a Fisher’s *post hoc*. The Kolmogorov-Smirnov test was used to compare cumulative sums. To compare the latencies among brief-access tests, we used a one-way ANOVA with a Fisher’s *post hoc*. The differences in the distributions (for lick rates in Total, Complete, Incomplete trials, and tastant/water ratio) in each column of Table 1 were tested, using Kruskal-Wallis. We used a Dunn's *post hoc* test for comparison among tasks. Data from the microestructural analysis of licking was compared across behavioral tests and Trial Types by performing a one-way ANOVA. The proportions of Palatability-related and Concentration-related neurons in each behavioral test, and proportions of licking-coherent neurons of each subset were compared among tasks using the chi-squared test. The values of coherence of licking-coherent neurons were compared between behavioral tests with a one-way ANOVA followed by a Fisher’s *post hoc*. We used a chi-squared test to compare proportions of Lick-Active and Lick-Inactive neurons across behavioral tests. The latencies of onsets of phasic inhibitions were tested with a one-way ANOVA with behavioral tests as a factor. The proportions of neuronal responses to auditory stimuli were compared among tasks using a chi-squared test (contingency table). Finally, the overall decoding performance during the Reward epoch was tested with a two-way ANOVA with behavioral tests as Level 1 (with 3 factors = the three brief access tests) and functional groups as Level 2 (with 5 factors) as independent variables. For all statistical tests, the alpha level was set at 0.05.


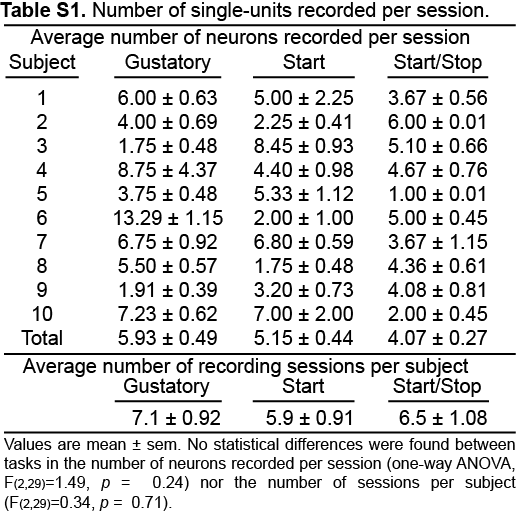


**Supplemental References**

Agresti, A. (2007). “Contingency Tables,” in *An Introduction to Categorical Data Analysis* (John Wiley & Sons, Inc.), 21–64. doi:10.1002/9780470114759.ch2.

| **Figures** | **Panels** | **Statistic test** |  | ***Post hoc* test** |  |
| --- | --- | --- | --- | --- | --- |
| 2 | B | Two-way ANOVA | Task factor: F(2,4)=911.5, *p* < 0.0001 | Fisher's LSD | Start vs. StartStop *p*<0.0001 |
|  |  | (Lick rate) | Trial Type factor: F(2,4)=1325.8, *p* < 0.0001 |  | Gustatory vs. StartStop *p*<0.0001 |
|  |  |  | Interaction task*trial type: F(8,29467)=18.8, *p* < 0.0001 |  | Gustatory vs.Start *p*<0.0001 |
|  |  |  |  |  | 0% vs. 3% *p*<0.0001 |
|  |  |  |  |  | 0% vs. 5.8%  *p*<0.0001 |
|  |  |  |  |  | 0% vs. 10.7% *p*<0.0001 |
|  |  |  |  |  | 0% vs. 20% *p*<0.0001 |
|  |  |  |  |  | 3% vs. 5.8% *p*<0.0001 |
|  |  |  |  |  | 3% vs. 10.7% *p*<0.0001 |
|  |  |  |  |  | 3% vs. 20% *p*<0.0001 |
|  |  |  |  |  | 5.8% vs. 10.7% *p*<0.0001 |
|  |  |  |  |  | 5.8% vs. 20% *p*<0.0001 |
|  |  |  |  |  | 10.7% vs. 20% *p*=0.0008 |
| 2 | C | Two-way ANOVA | Task factor: F(2,4)=686.5, *p* < 0.0001 | Fisher's LSD | Start vs. StartStop *p*<0.0001 |
|  |  | (First lick bout | Trial Type factor: F(2,4)=316.5, *p* < 0.0001 |  | Gustatory vs. StartStop *p*<0.0001 |
|  |  | duration) | Interaction task*trial type: F(8,29467)=16.4, *p* < 0.0001 |  | Gustatory vs.Start *p*<0.0001 |
|  |  |  |  |  | 0% vs. 3% *p*<0.0001 |
|  |  |  |  |  | 0% vs. 5.8%  *p*<0.0001 |
|  |  |  |  |  | 0% vs. 10.7% *p*<0.0001 |
|  |  |  |  |  | 0% vs. 20% *p*<0.0001 |
|  |  |  |  |  | 3% vs. 5.8% *p*<0.0001 |
|  |  |  |  |  | 3% vs. 10.7% *p*<0.0001 |
|  |  |  |  |  | 3% vs. 20% *p*<0.0001 |
|  |  |  |  |  | 5.8% vs. 10.7% *p*<0.0001 |
|  |  |  |  |  | 5.8% vs. 20% *p*<0.0001 |
|  |  |  |  |  | 10.7% vs. 20% *p*=0.725 |
| 2 | C | One-way ANOVA | Task factor: F(2,5917)=144.2, *p* < 0.0001 | Fisher's LSD | Gustatory vs.Start *p*<0.0001 |
|  |  | (Water rejection |  |  | Gustatory vs. StartStop *p*=0.002 |
|  |  | time) |  |  | Start vs. StartStop *p*<0.0001 |
| 2 | C | Chi-squared | Gustatory vs. Start χ2 (1, N=21055) = 0.79, *p* = 0.37 |  |  |
|  |  | (Propotions of single | Gustatory vs. Start/Stop χ2 (1, N=17440)= 4.09, *p* = 0.04 |  |  |
|  |  | lick bouts) | Start vs. Start/Stop χ2 (1, N=20469)= 8.16, *p* = 0.0043 |  |  |
| 3 | A | Two-way ANOVA | Task factor: F(2,4)=132.4, *p* < 0.0001 | Fisher's LSD | Start vs. StartStop *p*<0.0001 |
|  |  | (Bout index) | Trial Type factor: F(2,4)=92.3, *p* < 0.0001 |  | Gustatory vs. StartStop *p*=0.0002 |
|  |  |  | Interaction task*trial type: F(8,1530)=5.8, *p* < 0.0001 |  | Gustatory vs.Start *p*<0.0001 |
|  |  |  |  |  | 0% vs. 3% *p*<0.0001 |
|  |  |  |  |  | 0% vs. 5.8%  *p*<0.0001 |
|  |  |  |  |  | 0% vs. 10.7% *p*<0.0001 |
|  |  |  |  |  | 0% vs. 20% *p*<0.0001 |
|  |  |  |  |  | 3% vs. 5.8% *p*<0.0001 |
|  |  |  |  |  | 3% vs. 10.7% *p*<0.0001 |
|  |  |  |  |  | 3% vs. 20% *p*<0.0001 |
|  |  |  |  |  | 5.8% vs. 10.7% *p*<0.0001 |
|  |  |  |  |  | 5.8% vs. 20% *p*<0.0001 |
|  |  |  |  |  | 10.7% vs. 20% *p*=0.284 |
| 3 | B | Kolmogorov-Smirnov | Gustatory vs. Start D = 0.23, *p*=0.083 |  |  |
|  |  | (Cumulative sums of wet | Gustatory vs. Start/Stop D = 0.25, *p*=0.05 |  |  |
|  |  | licks) | Start vs. Start/Stop D = 0.41, *p*<0.0001 |  |  |
| 3 | C | Two-way ANOVA | Task factor: F(2,3)=72, *p* < 0.0001 | Fisher's LSD | Start vs. StartStop *p*=0.0002 |
|  |  | (Calorie intake) | Trial Type factor: F(2,3)=2730.3, *p* < 0.0001 |  | Gustatory vs. StartStop *p*<0.0001 |
|  |  |  | Interaction task*trial type: F(6,1176)=12.6, *p* < 0.0001 |  | Gustatory vs.Start *p*<0.0001 |
|  |  |  |  |  | 0% vs. 3% *p*<0.0001 |
|  |  |  |  |  | 0% vs. 5.8%  *p*<0.0001 |
|  |  |  |  |  | 0% vs. 10.7% *p*<0.0001 |
|  |  |  |  |  | 0% vs. 20% *p*<0.0001 |
|  |  |  |  |  | 3% vs. 5.8% *p*<0.0001 |
|  |  |  |  |  | 3% vs. 10.7% *p*<0.0001 |
|  |  |  |  |  | 3% vs. 20% *p*<0.0001 |
|  |  |  |  |  | 5.8% vs. 10.7% *p*<0.0001 |
|  |  |  |  |  | 5.8% vs. 20% *p*<0.0001 |
|  |  |  |  |  | 10.7% vs. 20% *p*<0.0001 |
| 3 | D | One-way ANOVA | Task factor: F(2,291)=46.5, *p* < 0.0001 | Fisher's LSD | Gustatory vs.Start *p*= 0.0002 |
|  |  | (Latency to stop licking |  |  | Gustatory vs. StartStop *p*<0.0001 |
|  |  | the empty sipper) |  |  | Start vs. StartStop *p*<0.0001 |
| 4 | C | Chi-squared | Gustatory vs. Start χ2 (1, N=230) = 2.12, *p* = 0.14 |  |  |
|  |  | (Proportions of Palatabi- | Gustatory vs. Start/Stop χ2 (1, N=188)= 0.56, *p* = 0.45 |  |  |
|  |  | lity-relaed neurons) | Start vs. Start/Stop χ2 (1, N=176)= 3.93, *p* = 0.047 |  |  |
| 4 | G | Chi-squared | Gustatory vs. Start χ2 (1, N=98) = 0.03, *p* = 0.86 |  |  |
|  |  | (Proportions of Concen- | Gustatory vs. Start/Stop χ2 (1, N=96)= 0.32, *p* = 0.57 |  |  |
|  |  | tration-related neurons) | Start vs. Start/Stop χ2 (1, N=82)= 0.14, *p* = 0.7 |  |  |
| 4 | C & G | Chi-squared | Palatability vs. Concentration χ2 (1, N=435) = 47.8, *p* < 0.001 |  |  |
| 6 | B | Chi-squared | Active Gustatory vs. Start χ2 (1, N=725) = 0.007, *p* = 0.9 |  |  |
|  |  | (Proportions of Lick-Ac- | Active Gustatory vs. Start/Stop χ2 (1, N=686)= 2.81, *p* = 0.09 |  |  |
|  |  | tive and Lick-Inactive | Active Start vs. Start/Stop χ2 (1, N=569)=2.63, *p* = 0.1 |  |  |
|  |  | neurons) | Inactive Gustatory vs. Start χ2 (1, N=725) = 0.018, *p* = 0.9 |  |  |
|  |  |  | Inactive Gustatory vs. Start/Stop χ2 (1, N=686)= 0.057, *p* = 0.8 |  |  |
|  |  |  | Inactive Start vs. Start/Stop χ2 (1, N=569)=0.118, *p* = 0.73 |  |  |
| 6 | C | Chi-squared | Active Gustatory vs. Start χ2 (1, N=725)=3.53, *p* = 0.06 |  |  |
|  |  | (Proportions of Lick-Ac- | Active Gustatory vs. Start/Stop χ2 (1, N=686)=0.21, *p* = 0.64 |  |  |
|  |  | tive and Lick-Inactive | Active Start vs. Start/Stop χ2 (1, N=569)=1.47, *p* = 0.22 |  |  |
|  |  | Start cue-responsive | Inactive Gustatory vs. Start χ2 (1, N=725)=44.7, *p* < 0.001 |  |  |
|  |  | neurons) | Inactive Gustatory vs. Start/Stop χ2 (1, N=686)=14.5, *p* < 0.001 |  |  |
|  |  |  | Inactive Start vs. Start/Stop χ2 (1, N=569)=6.4, *p* = 0.011 |  |  |
| 6 | D | Chi-squared | Inactive Gustatory vs. Start χ2 (1, N=96)=0.02, *p* = 0.9 |  |  |
|  |  | (Proportions of Lick-In- | Inactive Gustatory vs. Start/Stop χ2 (1, N=59)=6.72, *p* = 0.01 |  |  |
|  |  | active and Start and | Inactive Start vs. Start/Stop χ2 (1, N=113)=16.4, *p* < 0.001 |  |  |
|  |  | Stop cues responsive) |  |  |  |
| 7 | A | Two-way ANOVA | Task factor: F(2,4)=262.5, *p* < 0.0001 | Fisher's LSD | Start vs. StartStop  *p*<0.0001 |
|  |  | (Neuronal populations' | Functional ensemble factor: F(2,4)=215.9, *p* < 0.0001 |  | Gustatory vs. StartStop *p*<0.0001 |
|  |  | decoding performance | Interaction task*function ensemble: F(8,105)=20.79, *p* < 0.0001 |  | Gustatory vs.Start *p*<0.0001 |
|  |  | during the Reward |  |  | Active vs. Inactive  *p* <0.0001 |
|  |  | epoch) |  |  | Active vs. Coherent *p* <0.0001 |
|  |  |  |  |  | Active vs. Palatability *p* <0.0001 |
|  |  |  |  |  | Active vs. Concentration *p* <0.0001 |
|  |  |  |  |  | Inactive vs. Coherent *p* =0.0055 |
|  |  |  |  |  | Inactive vs. Palatability *p* <0.0001 |
|  |  |  |  |  | Inactive vs. Concentration *p* =0.0005 |
|  |  |  |  |  | Coherent vs. Palatability *p* <0.0001 |
|  |  |  |  |  | Coherent vs. Concentration *p* =0.46 |
|  |  |  |  |  | Palatability vs. Concentration *p* <0.0001 |
| 7 | C | Kruskal-wallis | Gustatory task |  |  |
|  |  | (Modulated-dropped | Modulated vs. Palatability: χ2 (2, N=340)=11.3, p = 0.0008 |  |  |
|  |  | neurons decoding per- | Modulated vs. Concentration: χ2 (2, N=340)=0, p = 1 |  |  |
|  |  | formance during the | Modulated vs. Coherent: χ2 (2, N=340)=0, p = 1 |  |  |
|  |  | Reward epoch) | Modulated vs. Lick-Active: χ2 (2, N=340)=1.33, p = 0.248 |  |  |
|  |  |  | Modulated vs. Lick-Inactive: χ2 (2, N=340)=9.28, p = 0.0023 |  |  |
|  |  |  | Start task |  |  |
|  |  |  | Modulated vs. Palatability: χ2 (2, N=340)=11.29, p = 0.0008 |  |  |
|  |  |  | Modulated vs. Concentration: χ2 (2, N=340)=0.4, p = 0.53 |  |  |
|  |  |  | Modulated vs. Coherent: χ2 (2, N=340)=0.1, p = 0.75 |  |  |
|  |  |  | Modulated vs. Lick-Active: χ2 (2, N=340)=2.48, p = 0.11 |  |  |
|  |  |  | Modulated vs. Lick-Inactive: χ2 (2, N=340)=4.41, p = 0.0357 |  |  |
|  |  |  | Start/Stop task |  |  |
|  |  |  | Modulated vs. Palatability: χ2 (2, N=340)=11.3, p = 0.0008 |  |  |
|  |  |  | Modulated vs. Concentration: χ2 (2, N=340)=0.04, p = 0.83 |  |  |
|  |  |  | Modulated vs. Coherent: χ2 (2, N=340)=3.19, p = 0.074 |  |  |
|  |  |  | Modulated vs. Lick-Active: χ2 (2, N=340)=3.19, p = 0.074 |  |  |
|  |  |  | Modulated vs. Lick-Inactive: χ2 (2, N=340)=8.65, p = 0.0033 |  |  |
| 9 | B | Chi-squared | Gustatory vs. Start χ2 (1, N=725)=7.6, *p* = 0.006 |  |  |
|  |  | (Proportions of phasic | Gustatory vs. Start/Stop χ2 (1, N=686)=1.62, *p* = 0.2 |  |  |
|  |  | inhibitory neurons) | Start vs. Start/Stop χ2 (1, N=569)=13.5, *p* < 0.001 |  |  |
| 9 | B | One-way ANOVA | Task factor: F(2,47)=6, *p* =0.0048 | Fisher's LSD | Gustatory vs.Start *p* = 0.17 |
|  |  | (Phasic inhibitions at RE |  |  | Gustatory vs. StartStop *p* = 0.01 |
|  |  | onset latencies) |  |  | Start vs. StartStop *p* = 0.0048 |
| Table 1 | Column 1 | Kruskal-wallis | Task factor: χ2 (2, N=5919)=435.99, p < 0.0001 | Dunn's test | Gustatory vs.Start *p* < 0.0001 |
|  | Total | (Lick rate 0 %) |  |  | Gustatory vs. StartStop *p* < 0.0001 |
|  |  |  |  |  | Start vs. StartStop *p* < 0.0001 |
| Table 1 | Column 2 | Kruskal-wallis | Task factor: χ2 (2, N=5892)=220.19, p < 0.0001 | Dunn's test | Gustatory vs.Start *p* = 0.011 |
|  | Total | (Lick rate 3 %) |  |  | Gustatory vs. StartStop *p* < 0.0001 |
|  |  |  |  |  | Start vs. StartStop *p* < 0.0001 |
| Table 1 | Column 3 | Kruskal-wallis | Task factor: χ2 (2, N=5886)=315.31, p < 0.0001 | Dunn's test | Gustatory vs.Start *p*  = 0.0096 |
|  | Total | (Lick rate 5.8 %) |  |  | Gustatory vs. StartStop *p* < 0.0001 |
|  |  |  |  |  | Start vs. StartStop *p* < 0.0001 |
| Table 1 | Column 4 | Kruskal-wallis | Task factor: χ2 (2, N=5919)=523.39, p < 0.0001 | Dunn's test | Gustatory vs.Start *p* = 0.98 |
|  | Total | (Lick rate 10.7 %) |  |  | Gustatory vs. StartStop *p* < 0.0001 |
|  |  |  |  |  | Start vs. StartStop *p* < 0.0001 |
| Table 1 | Column 5 | Kruskal-wallis | Task factor: χ2 (2, N=5896)=708.48, p < 0.0001 | Dunn's test | Gustatory vs.Start *p* < 0.0001 |
|  | Total | (Lick rate 20 %) |  |  | Gustatory vs. StartStop *p* < 0.0001 |
|  |  |  |  |  | Start vs. StartStop *p* < 0.0001 |
| Table 1 | Column 1 | Kruskal-wallis | Task factor: χ2 (2, N=926)=37.57, p < 0.0001 | Dunn's test | Gustatory vs.Start *p* = 0.58 |
|  | Complete | (Lick rate 0 %) |  |  | Gustatory vs. StartStop *p* < 0.0001 |
|  |  |  |  |  | Start vs. StartStop *p* = 0.0006 |
| Table 1 | Column 2 | Kruskal-wallis | Task factor: χ2 (2, N=1419)=78.41, p < 0.0001 | Dunn's test | Gustatory vs.Start *p* < 0.0001 |
|  | Complete | (Lick rate 3 %) |  |  | Gustatory vs. StartStop *p* < 0.0001 |
|  |  |  |  |  | Start vs. StartStop *p* < 0.0001 |
| Table 1 | Column 3 | Kruskal-wallis | Task factor: χ2 (2, N=1889)=141.8, p < 0.0001 | Dunn's test | Gustatory vs.Start *p*  < 0.0001 |
|  | Complete | (Lick rate 5.8 %) |  |  | Gustatory vs. StartStop *p* < 0.0001 |
|  |  |  |  |  | Start vs. StartStop *p* < 0.0001 |
| Table 1 | Column 4 | Kruskal-wallis | Task factor: χ2 (2, N=2520)=200.75, p < 0.0001 | Dunn's test | Gustatory vs.Start *p* = 0.37 |
|  | Complete | (Lick rate 10.7 %) |  |  | Gustatory vs. StartStop *p* < 0.0001 |
|  |  |  |  |  | Start vs. StartStop *p* < 0.0001 |
| Table 1 | Column 5 | Kruskal-wallis | Task factor: χ2 (2, N=2416)=244.8, p < 0.0001 | Dunn's test | Gustatory vs.Start *p* < 0.0001 |
|  | Complete | (Lick rate 20 %) |  |  | Gustatory vs. StartStop *p* < 0.0001 |
|  |  |  |  |  | Start vs. StartStop *p* < 0.0001 |
| Table 1 | Column 1 | Kruskal-wallis | Task factor: χ2 (2, N=256.33)=4992, p < 0.0001 | Dunn's test | Gustatory vs.Start *p* < 0.0001 |
|  | Incomplete | (Lick rate 0 %) |  |  | Gustatory vs. StartStop *p* < 0.0001 |
|  |  |  |  |  | Start vs. StartStop *p* < 0.0001 |
| Table 1 | Column 2 | Kruskal-wallis | Task factor: χ2 (2, N=4472)=90.15, p < 0.0001 | Dunn's test | Gustatory vs.Start *p* = 0.0076 |
|  | Incomplete | (Lick rate 3 %) |  |  | Gustatory vs. StartStop *p* < 0.0001 |
|  |  |  |  |  | Start vs. StartStop *p* < 0.0001 |
| Table 1 | Column 3 | Kruskal-wallis | Task factor: χ2 (2, N=3996)=107.38, p < 0.0001 | Dunn's test | Gustatory vs.Start *p* < 0.0001 |
|  | Incomplete | (Lick rate 5.8 %) |  |  | Gustatory vs. StartStop *p* < 0.0001 |
|  |  |  |  |  | Start vs. StartStop *p* < 0.0001 |
| Table 1 | Column 4 | Kruskal-wallis | Task factor: χ2 (2, N=3363)=75.88, p < 0.0001 | Dunn's test | Gustatory vs.Start *p* < 0.0001 |
|  | Incomplete | (Lick rate 10.7 %) |  |  | Gustatory vs. StartStop *p* < 0.0001 |
|  |  |  |  |  | Start vs. StartStop *p* < 0.0001 |
| Table 1 | Column 5 | Kruskal-wallis | Task factor: χ2 (2, N=3479)=64.51, p < 0.0001 | Dunn's test | Gustatory vs.Start *p* < 0.0001 |
|  | Incomplete | (Lick rate 20 %) |  |  | Gustatory vs. StartStop *p* < 0.0001 |
|  |  |  |  |  | Start vs. StartStop *p* = 0.0064 |
| Table 1 | Column 2 | Kruskal-wallis | Task factor: χ2 (2, N=296)=74.24, p < 0.0001 | Dunn's test | Gustatory vs.Start *p* < 0.0001 |
|  |  | (Tastant/water ratio |  |  | Gustatory vs. StartStop *p* = 0.0003 |
|  |  | 3 %) |  |  | Start vs. StartStop *p* < 0.0001 |
| Table 1 | Column 3 | Kruskal-wallis | Task factor: χ2 (2, N=296)=63.84, p < 0.0001 | Dunn's test | Gustatory vs.Start *p* < 0.0001 |
|  |  | (Tastant/water ratio |  |  | Gustatory vs. StartStop *p* = 0.0084 |
|  |  | 5.8 %) |  |  | Start vs. StartStop *p* < 0.0001 |
| Table 1 | Column 4 | Kruskal-wallis | Task factor: χ2 (2, N=296)=52.68, p < 0.0001 | Dunn's test | Gustatory vs.Start *p* < 0.0001 |
|  |  | (Tastant/water ratio |  |  | Gustatory vs. StartStop *p* = 0.0545 |
|  |  | 10.7 %) |  |  | Start vs. StartStop *p* < 0.0001 |
| Table 1 | Column 5 | Kruskal-wallis | Task factor: χ2 (2, N=296)=38.67, p < 0.0001 | Dunn's test | Gustatory vs.Start *p* < 0.0001 |
|  |  | (Tastant/water ratio |  |  | Gustatory vs. StartStop *p* = 0.17 |
|  |  | 20 %) |  |  | Start vs. StartStop *p* < 0.0001 |
| Table 2 | Row 1 | One-way ANOVA | Task factor: F(2,5841)=91.8, *p <* 0.0001 | Dunn's test | Gustatory vs.Start *p* < 0.0001 |
|  |  | (Number of lick bouts |  |  | Gustatory vs. StartStop *p <* 0.0001 |
|  |  | for 0 % sucrose) |  |  | Start vs. StartStop *p* < 0.0001 |
| Table 2 | Row 2 | One-way ANOVA | Task factor: F(2,5815)=165.7, *p <* 0.0001 | Dunn's test | Gustatory vs.Start *p =* 0.18 |
|  |  | (Number of lick bouts |  |  | Gustatory vs. StartStop  *p <* 0.0001 |
|  |  | for 3 % sucrose) |  |  | Start vs. StartStop *p* < 0.0001 |
| Table 2 | Row 3 | One-way ANOVA | Task factor: F(2,5838)=218.1, *p <* 0.0001 | Dunn's test | Gustatory vs.Start *p* = 0.99 |
|  |  | (Number of lick bouts |  |  | Gustatory vs. StartStop  *p <* 0.0001 |
|  |  | for 5.8 % sucrose) |  |  | Start vs. StartStop *p* < 0.0001 |
| Table 2 | Row 4 | One-way ANOVA | Task factor: F(2,5839)=186.2, *p <* 0.0001 | Dunn's test | Gustatory vs.Start *p* < 0.0001 |
|  |  | (Number of lick bouts |  |  | Gustatory vs. StartStop *p <* 0.0001 |
|  |  | for 10.7 % sucrose) |  |  | Start vs. StartStop *p* < 0.0001 |
| Table 2 | Row 5 | One-way ANOVA | Task factor: F(2,5847)=228.4, *p <* 0.0001 | Dunn's test | Gustatory vs.Start *p =* 0.99 |
|  |  | (Number of lick bouts |  |  | Gustatory vs. StartStop  *p <* 0.0001 |
|  |  | for 20 % sucrose) |  |  | Start vs. StartStop *p* < 0.0001 |
| Table 2 | Row 1 | One-way ANOVA | Task factor: F(2,10353)=172.3, *p <* 0.0001 | Dunn's test | Gustatory vs.Start *p* < 0.0001 |
|  |  | (Duration of lick bouts |  |  | Gustatory vs. StartStop *p <* 0.0001 |
|  |  | for 0 % sucrose) |  |  | Start vs. StartStop *p* < 0.0001 |
| Table 2 | Row 2 | One-way ANOVA | Task factor: F(2,11914)=198.3, *p <* 0.0001 | Dunn's test | Gustatory vs.Start *p* = 0.18 |
|  |  | (Duration of lick bouts |  |  | Gustatory vs. StartStop  *p <* 0.0001 |
|  |  | for 3 % sucrose) |  |  | Start vs. StartStop *p* < 0.0001 |
| Table 2 | Row 3 | One-way ANOVA | Task factor: F(2,12633)=255.9, *p <* 0.0001 | Dunn's test | Gustatory vs.Start *p* = 0.91 |
|  |  | (Duration of lick bouts |  |  | Gustatory vs. StartStop  *p <* 0.0001 |
|  |  | for 5.8 % sucrose) |  |  | Start vs. StartStop *p* < 0.0001 |
| Table 2 | Row 4 | One-way ANOVA | Task factor: F(2,11774)=257.1, *p <* 0.0001 | Dunn's test | Gustatory vs.Start *p* < 0.0001 |
|  |  | (Duration of lick bouts |  |  | Gustatory vs. StartStop *p <* 0.0001 |
|  |  | for 10.7 % sucrose) |  |  | Start vs. StartStop *p* < 0.0001 |
| Table 2 | Row 5 | One-way ANOVA | Task factor: F(2,11994)=303.2, *p <* 0.0001 | Dunn's test | Gustatory vs.Start *p* = 0.12 |
|  |  | (Duration of lick bouts |  |  | Gustatory vs. StartStop  *p <* 0.0001 |
|  |  | for 20 % sucrose) |  |  | Start vs. StartStop *p* < 0.0001 |
| Table 2 | Column 1 | One-way ANOVA | Task factor: F(2,8921)=19.86, *p <* 0.0001 | Dunn's test | 0 vs. 3 % *p* < 0.0001 |
|  |  | (Number of lick bouts |  |  | 0 vs. 5.8 % *p <* 0.0001 |
|  |  | in the Gustatory task) |  |  | 0 vs. 10.7 % *p* < 0.0001 |
|  |  |  |  |  | 0 vs. 20 % *p* < 0.0001 |
| Table 2 | Column 2 | One-way ANOVA | Task factor: F(2,19921)=100.5, *p <* 0.0001 | Dunn's test | 0 vs. 3 % *p* = 0.83 |
|  |  | (Duration of lick bouts |  |  | 0 vs. 5.8 % *p <* 0.0001 |
|  |  | in the Gustatory task) |  |  | 0 vs. 10.7 % *p* < 0.0001 |
|  |  |  |  |  | 0 vs. 20 % *p* < 0.0001 |
| Table 2 | Column 3 | One-way ANOVA | Task factor: F(2,11932)=89.12, *p <* 0.0001 | Dunn's test | 0 vs. 3 % *p* < 0.0001 |
|  |  | (Number of lick bouts |  |  | 0 vs. 5.8 % *p <* 0.0001 |
|  |  | in the Start task) |  |  | 0 vs. 10.7 % *p* < 0.0001 |
|  |  |  |  |  | 0 vs. 20 % *p* < 0.0001 |
| Table 2 | Column 4 | One-way ANOVA | Task factor: F(2,25857)=361.09, *p <* 0.0001 | Dunn's test | 0 vs. 3 % *p* < 0.0001 |
|  |  | (Duration of lick bouts |  |  | 0 vs. 5.8 % *p <* 0.0001 |
|  |  | in the Start task) |  |  | 0 vs. 10.7 % *p* < 0.0001 |
|  |  |  |  |  | 0 vs. 20 % *p* < 0.0001 |
| Table 2 | Column 5 | One-way ANOVA | Task factor: F(2,8329)=3.14, *p*= 0.0137 | Dunn's test | 0 vs. 3 % *p* = 0.32 |
|  |  | (Number of lick bouts |  |  | 0 vs. 5.8 % *p =*0.29 |
|  |  | in the Start/Stop task) |  |  | 0 vs. 10.7 % *p* = 0.99 |
|  |  |  |  |  | 0 vs. 20 % *p = 0.99* |
| Table 2 | Column 6 | One-way ANOVA | Task factor: F(2,12892)=257.77, *p*< 0.0001 | Dunn's test | 0 vs. 3 % *p* < 0.0001 |
|  |  | Duration of lick bouts |  |  | 0 vs. 5.8 % *p <* 0.0001 |
|  |  | in the Start/Stop task) |  |  | 0 vs. 10.7 % *p* < 0.0001 |
|  |  |  |  |  | 0 vs. 20 % *p* < 0.0001 |
| Table 3 | All coherent | Chi-squared | Gustatory vs. Start χ2 (1, N=725) = 0.33, *p* = 0.56 |  |  |
|  | neurons | (Proportions of | Gustatory vs. Start/Stop χ2 (1, N=686)= 0.6, *p* = 0.44 |  |  |
|  |  | neurons) | Start vs. Start/Stop χ2 (1, N=569)= 0.04, *p* = 0.83 |  |  |
| Table 3 | All coherent | One-way ANOVA | Task factor: F(2,83)=3.86, *p* =0.0251 | Fisher's LSD | Gustatory vs.Start *p* = 0.118 |
|  | neurons | (Coherence coefficients) |  |  | Gustatory vs. StartStop *p* = 0.18 |
|  |  |  |  |  | Start vs. StartStop *p* = 0.0069 |
